# External Apical Root Resorption with Clear Aligners Versus Fixed Appliances: A Scoping Review of Comparative Evidence, Treatment-Related Factors, Imaging Modalities and Consistency of Reported Data

**DOI:** 10.3390/jcm15218219

**Published:** 2026-10-22

**Authors:** Bianca Luminița (Erhan) Groza, Emilia (Prodea) Rusu, Mariana Păcurar, Maria Popa, Mihai Dan Roman, Adrian-Gheorghe Boicean, Gabriela Bota, Laura Stef, Anca Maria Fratila, Denisa Elena Redean, Sorin Radu Fleacă

**Affiliations:** 1 Doctoral School, Faculty of Medicine, Lucian Blaga University of Sibiu, 2A Lucian Blaga Street, 550169 Sibiu, Romaniadenisaelena.redean@ulbsibiu.ro (D.E.R.); 2 Doctoral School, George Emil Palade University of Medicine, Pharmacy, Science, and Technology of Targu Mures, 38 Gheorghe Marinescu Street, 540139 Targu Mures, Romania; 3 Department of Orthodontics, George Emil Palade University of Medicine, Pharmacy, Science, and Technology of Targu Mures, 38 Gheorghe Marinescu Street, 540139 Targu Mures, Romania; 4 Department of Orthodontics, Faculty of Medicine, Lucian Blaga University of Sibiu, 2A Lucian Blaga Street, 550169 Sibiu, Romania; amaria.popa@ulbsibiu.ro; 5 Department of Surgery, Faculty of Medicine, Lucian Blaga University of Sibiu, 2A Lucian Blaga Street, 550169 Sibiu, Romania; mihai.roman@ulbsibiu.ro; 6 Clinical Medical Department, Faculty of Medicine, Lucian Blaga University of Sibiu, 2A Lucian Blaga Street, 550169 Sibiu, Romania; adrian.boicean@ulbsibiu.ro; 7 Department of Dental Medicine and Nursing, Faculty of Medicine, Lucian Blaga University of Sibiu, 2A Lucian Blaga Street, 550169 Sibiu, Romania; gabriela.bota@ulbsibiu.ro (G.B.); laura.stef@ulbsibiu.ro (L.S.); anca.fratila@ulbsibiu.ro (A.M.F.); 8 Clinical Surgical Department, Faculty of Medicine, Lucian Blaga University of Sibiu, 2A Lucian Blaga Street, 550169 Sibiu, Romania; radu.fleaca@ulbsibiu.ro

**Keywords:** root resorption, clear aligners, fixed orthodontic appliances, orthodontic tooth movement, cone-beam computed tomography, data consistency, evidence synthesis, scoping review

## Abstract

Most meta-analyses report less external apical root resorption (EARR) with clear aligners (CAs) than fixed appliances (FAs), but pooled differences are sub-millimetric and rest on few recurring studies. This scoping review (PRISMA-ScR) mapped how 2020–2026 evidence on CA–FA comparisons, treatment-related factors and imaging modalities was generated, and checked data consistency, without pooling. Its unregistered protocol (version 1.0) was reconstructed during manuscript preparation. PubMed, Scopus and Web of Science were searched; paired reviewers screened 1688 records and 263 full texts and included 176 reports (174 studies). Fifteen reports, including eight of 32 direct-comparison reports, were excluded because outcome data were not attributable to a distinct sample or not identifiable; in one, pooled by six syntheses, aligner values equal another study’s fixed-appliance values. Of 21 included comparisons, 17 had serious or high risk of bias, one accounted for clustering and three reported measurement error in millimetres; differences were 0.01–1.12 mm except in one. Fifteen of 17 studies not described as randomised reported less EARR with CAs; the only concealed-allocation trial of appliances alone found no difference. Two imaging comparisons disagreed on whether panoramic radiography under- or overestimates EARR. Differences should be judged against measurement error and design, and data consistency should be checked before pooling.

## 1. Introduction

External apical root resorption (EARR) is an almost universal histological consequence of orthodontic tooth movement, whereas clinically relevant root shortening affects a minority of teeth and patients and is influenced by both treatment-related and patient-related factors [1,2]. It is a response of the periodontal tissues to orthodontic force: compression of the periodontal ligament and the ensuing clastic activity remove cementum and, subsequently, dentine at the root apex, and because resorbed apical structure is not regenerated, EARR remains one of the complications most frequently discussed when patients consent to orthodontic treatment [1].

Clear aligners (CAs) have been hypothesised to cause less EARR than fixed appliances (FAs) because the force delivered by each aligner decays within a treatment stage and because aligners are removed for part of the day. The comparison has been meta-analysed repeatedly since 2019 [3,4,5,6,7,8,9]: in non-extraction cases [4], in a review of 10 comparative studies published up to July 2023, six of which were pooled [5], in comparative studies of incisors [6] and in studies restricted to cone-beam computed tomography (CBCT) [7,8,9]; earlier reviews have been summarised in an umbrella review [10] and in an overview that rated the four systematic reviews it included, published up to 2022, as of low or critically low methodological quality [11]. Most of these syntheses report less EARR with CAs, although a CBCT-based meta-analysis of five studies [8] and the meta-analysis of non-extraction cases [4] found no significant difference between appliances, except for one maxillary lateral incisor in the latter. Where a difference is reported, it is of the order of a few tenths of a millimetre, and the same small set of primary studies, typically retrospective CBCT cohorts [12], recurs across the syntheses; a 2026 comparative meta-analysis pooled four such cohorts [9].

Differences of this magnitude are difficult to interpret without considering how they were obtained. Two-dimensional radiographs misclassify apical root loss relative to CBCT [13,14]; the measurement error of the methods used is rarely reported in millimetres; and studies differ in the landmarks used, in the outcome metric (linear, volumetric or ratio-based), in whether teeth clustered within patients are analysed as independent observations, and in whether case complexity, extraction, treatment duration and the type and amount of tooth movement are controlled. Movement type itself is associated with EARR [15], and some comparative studies assess a single movement phase rather than comprehensive treatment [16]. Pooled estimates that do not account for these features may combine measurements that are not comparable.

A further, largely unexamined consideration is the consistency of the data reported by the underlying studies. Evidence syntheses increasingly check whether the data of included studies are unique and internally consistent, for example, whether outcome values recur in other publications or whether the reported numbers agree with each other, because such discrepancies can distort pooled results [17,18]. Publication of the same data more than once without cross-reference has been shown to inflate pooled treatment effects [19,20], and when a trustworthiness screening tool was applied as an inclusion criterion to 18 Cochrane reviews, a quarter of their trials were reclassified and six of the reviews were judged to require updating [21]. Risk-of-bias tools, which assess how a study was designed and conducted, are not designed to detect whether its data are unique or internally consistent. When the effect at stake is a few tenths of a millimetre, a single dataset counted twice or reported inconsistently can materially change a pooled estimate.

These questions concern how the evidence was generated rather than the size of an effect, and they span three related bodies of evidence: direct CA–FA comparisons, studies of treatment factors associated with EARR, and studies comparing imaging modalities. A scoping review is therefore the appropriate design, because its purpose is to map the characteristics of the available evidence and to identify gaps, not to estimate an effect or to support clinical recommendations [22,23]. As the use of scoping reviews in orthodontics has been questioned when the underlying question is one of effectiveness [24], we state explicitly that this review does not pool results, does not rank appliances and makes no clinical recommendation on appliance selection. Its contributions are a map of how EARR is measured and analysed across the recent clinical literature and a check of the consistency of the reported data in this evidence base.

For clinical studies published between 1 January 2020 and 1 September 2026, the review addressed three questions: (1) What are the characteristics and reported findings of studies directly comparing EARR between CAs and FAs? (2) Which treatment-related factors—movement type, extraction, treatment duration or other treatment factors—have been analysed in relation to EARR in CA or FA studies, and with which designs and imaging modalities? (3) What evidence exists on differences in EARR quantification between imaging modalities?

## 2. Materials and Methods

### 2.1. Design, Reporting Guideline and Protocol

This scoping review followed the JBI methodology for scoping reviews [23], which builds on the framework of Arksey and O’Malley [25] as refined by Levac et al. [26], and is reported according to the PRISMA extension for scoping reviews (PRISMA-ScR) [27]; the completed checklist is provided as Table S1. A protocol (version 1.0), reconstructed during manuscript preparation because no dated copy was retained, is provided in Table S3, Part A; it is undated, as no contemporaneous document records when it was written. Before the searches of 3 September 2026, its scope was revised to the three review questions stated above, to patients treated with either appliance, to a 2020–2026 publication window and to three databases, and the data-consistency criteria of Section 2.6 were added (version 1.1). Eight clarifications of the eligibility criteria were documented after full-text assessment to record how borderline cases had been handled (version 1.2, 25 September 2026); no selection decision was changed as a result. The two operational rules of Section 2.6 were made explicit during manuscript preparation (version 1.3, 26 September 2026) after a consistency check of all full-text decisions against them. The check changed three decisions (one report excluded under criterion (ii) and two under criterion (i)) and reassigned four included direct comparisons from Question 2 to Question 1. A second check, of every full-text decision against the full text, the clarifications and the two operational rules, changed 15 further decisions—13 reports previously excluded (eight for lack of an analysed comparison or of a quantitative apical outcome and five under criterion (ii)) were included and two were excluded under criterion (i)—reassigned one report from Question 2 to Question 1, corrected five exclusion codes without change of decision and added two clarifications (version 1.4, 27 September 2026). Both checks were performed independently by two authors (D.E.R., L.S.), and each changed decision was confirmed by S.R.F. All counts reported here reflect these decisions. All amendments and every deviation from the protocol, with reasons, are recorded in Table S3, Parts B and C. The protocol was not registered in a public registry, and we regard this, together with the reconstruction of its initial version, as a major methodological weakness of the review (Section 4.6).

Table S3a separates the elements defined before screening—the three review questions, the Population–Concept–Context criteria, the databases and search strings, the 18 predefined exclusion reasons, the two data-consistency criteria in their general form and the screening procedure—from the clarifications and operational rules introduced after full-text assessment, with the number of decisions affected by each. Post hoc decision-making was limited in two ways: each clarification and operational rule was written as a general rule and applied to all 263 full-text reports, not only to the report that prompted it, and every changed decision is listed in Table S3 with its record identifier, the rule applied and the direction of the change. The 18 changes went in both directions (13 inclusions and five exclusions); five concerned direct CA–FA comparisons (two inclusions and three exclusions; Table S13, scenario v) and 13 concerned Question 2 reports. The direction of effect of the direct comparisons under the decisions taken before the two checks is reported in Section 4.3.

Consistent with the purpose of a scoping review, no meta-analysis was performed [22,23]; the risk of bias was assessed only for the direct comparisons, to qualify their interpretation (Section 2.8).

### 2.2. Eligibility Criteria

Eligibility was defined with the Population–Concept–Context framework (Table 1). The publication window was chosen to map the current comparative evidence. It begins after the search periods of the earliest meta-analyses of the comparison, which included no study published from 2020 onwards [3,4], and it contains most of the studies pooled by each of the comparative syntheses published since 2024 (from 6 of 10 to 5 of 6 [5,6,7,8,9,10]). Studies published before 2020 were not mapped (Section 4.6). Reports were classified against a predefined list of 18 exclusion reasons (Table S4), one of which was recorded as the primary reason for each excluded report. A report was assigned to Question 1 when it gave EARR values separately for a CA group and an FA group treated in the same study, with a between-group comparison as a stated aim or a reported test, whether the appliance was its primary or a secondary factor; reports that entered appliance type only as a covariate, without group values, were assigned to Question 2. Single-cohort reports satisfied the concept of an analysed treatment factor only when EARR was related to at least one treatment-related variable (treatment duration, amount or type of tooth movement, protocol) by comparison, correlation or regression.

### 2.3. Information Sources and Search

PubMed/MEDLINE, Scopus and Web of Science Core Collection (all editions) were searched on 3 September 2026 with database-specific strategies combining controlled-vocabulary and free-text terms for root resorption with terms for orthodontic treatment, without filters for study design, language or article type; the strategies as run are provided in Table S2, and the search is reported in accordance with PRISMA-S [28] (checklist in Table S11). Publication-year limits of 2020–2026 were written into each strategy with the database’s own date field (PubMed publication date, Scopus PUBYEAR, Web of Science PY), and the PubMed strategy additionally excluded records indexed only to animals. The publication date of a record was defined as its earliest date of publication (online/early-access or print), and records with an earliest date after 1 September 2026 were removed before screening. The searches retrieved 850 (PubMed), 1221 (Scopus) and 1284 (Web of Science) records. No additional sources (reference lists, citation tracking, trial registries or grey literature) were searched.

### 2.4. Selection of Sources of Evidence

Records were imported into Zotero (version 9.0.6; Corporation for Digital Scholarship, Vienna, VA, USA), which was used for the initial deduplication, screening, record management and full-text retrieval. Duplicates were initially identified with the duplicate-detection function of Zotero and were not reviewed manually. During the revision, the deduplication was repeated on the original exports with a script that matched records on DOI, PubMed identifier and normalised title, in the order Web of Science, Scopus, PubMed, and every group of matched duplicates and every record without a DOI was reviewed individually; 44 records, almost all without a DOI, had been matched as duplicates but were distinct, and they were screened with the same procedure as the other records (Table S3, Part C). Screening started on 3 September 2026. Titles and abstracts were screened independently by two reviewers: the 10 screening authors were organised in five fixed pairs (B.L.(E.)G. and E.(P.)R.; M.Pă. and M.Po.; M.D.R. and A.-G.B.; G.B. and L.S.; A.M.F. and D.E.R.), and the records were allocated at random to the pairs in blocks of approximately equal size. Records judged potentially eligible by either reviewer were retained. Disagreements were resolved by an eleventh reviewer (S.R.F.), whose decision was final. Full texts of the retained records were sought as described in Section 2.5 and assessed for eligibility by the same pairs, again independently and with the same arbitration, between 11 and 20 September 2026; the 17 reports added during the revision (three identified at the repeated deduplication and 14 obtained during the revision; Section 2.5) were assessed in October 2026 with the clarifications and operational rules already in place (versions 1.2–1.4). For each report assessed at full text, the reviewers recorded the decision, the primary exclusion reason (Table S4) and notes documenting the data-consistency checks described in Section 2.6. Reports describing the same trial or cohort with a declared relationship were retained and linked as one study (Table S8).

### 2.5. Retrieval of Full Texts

Full texts were sought from PubMed Central, from publisher websites (subscription journals through the institutional access agreements of the Lucian Blaga University of Sibiu and the George Emil Palade University of Medicine, Pharmacy, Science, and Technology of Targu Mures), from the Unpaywall database (an index of open access copies of articles legally hosted by publishers and repositories) and with the “Find Full Text” function of Zotero; legal open access versions were downloaded manually when automated retrieval failed. Each retrieved file was checked for identity (title, authors, journal, DOI) and completeness. At the initial retrieval, corresponding authors were not contacted and interlibrary loan was not used. The 22 reports that could not be obtained through the institutional access of the two universities or as open access versions (19 in subscription journals and three no longer freely available) are listed with reasons in Table S5. During the revision (October 2026), retrieval of these reports was attempted again through an extension of the institutional access agreement, through interlibrary loan and by contacting their corresponding authors; 14 were obtained and assessed at full text with the same procedure, and the eight that could not be obtained, all in subscription journals, remain listed in Table S5.

### 2.6. Data-Consistency Checks

Two data-consistency criteria were applied as eligibility criteria at full text, adapted from current Cochrane guidance [17]: (i) outcome data and/or methods text identical or near-identical to those of another publication, without a shared cohort stated in either report, and (ii) values reported in different parts of the report that cannot be reconciled, so that the primary EARR outcome cannot be identified. The two criteria correspond to two items common to the checklists developed for randomised trials—overlap with other publications [19,20,29] and numbers that do not agree with each other [18,30]—and were chosen because they can be assessed from the published record of studies of any design, whereas the other items of those tools (governance, recruitment timeframe, attrition, baseline distributions) presuppose a randomised trial, a minority design in this evidence base. For each report, the reviewers (a) recalculated between-time-point differences from the reported pre- and post-treatment values, checked the consistency of sample sizes across the text, tables and figures and noted dispersion that was small relative to the stated measurement resolution; (b) compared outcome values, acquisition parameters and methods text with those of the other retrieved reports and of the main sources cited in each report; and (c) recorded any shared cohort stated in the report.

Two operational rules were applied uniformly. First, when the same research group reported near-identical values for groups described as independent in two or more reports with overlapping recruitment periods and no shared cohort stated, the earliest available report was retained and the later reports were excluded under criterion (i); the same rule applied to reports by different authors that reported values identical to those of an earlier publication. Second, criterion (ii) was met only when the primary EARR outcome could not be identified from the report (for example, category counts exceeding the stated group sizes, or two incompatible values for the same outcome); all other discrepancies—a subgroup value repeated in an abstract, conclusions that differ from the tabulated data, *p*-values or test statistics not reproducible from the printed summary statistics, standard deviations smaller than the measurement resolution, or differences between the numbers randomised and analysed not explained in the report—were recorded but did not lead to exclusion. Reports meeting criterion (i) or (ii) were excluded, and the observations supporting each decision, with page and table locators, are documented in Table S9, together with the discrepancies recorded in retained reports. These criteria concern only whether the reported data could be used to map the evidence; they imply no judgement about the quality of the reports or the conduct of the research, and they were not used as an appraisal of methodological quality. Readers can verify every observation in Table S9 and classify the reports differently. The Cochrane guidance [17] also provides for seeking clarification from the study authors and, where concerns remain, from the editor of the journal concerned; this review departs from that guidance, because the criteria describe only the published values and make no claim about how the data were generated. A report for which a correction or clarification is published would be reassessed under the same criteria. Deviations from the protocol in this procedure are listed in Table S3 (Part C).

### 2.7. Data Charting

Data were charted with a predefined form (Table S6) covering study design; country; sample size per group; age; malocclusion; extraction status; appliance systems; treatment phase and duration; teeth and roots assessed; imaging modality and acquisition parameters (voxel size, field of view); EARR metric and landmarks; definition of severe EARR; examiner blinding; reliability statistics; unit of analysis and handling of the clustering of teeth within patients; control of case complexity; and the reported EARR values and between-group differences. Each included report was assigned to one or more review questions. For the reports addressing Questions 1 and 3, all items were charted from the full texts, and every charted value was verified against the source PDF by B.L.(E.)G. and E.(P.)R.; for the reports addressing Question 2, design, setting, sample, appliance, analysed factor, imaging modality and EARR metric were charted at a lower level of detail and verified against the full text by the reviewer pair that had assessed each report. For comparative studies, the EARR outcome declared as primary in the report was identified; where none was declared, the principal EARR outcome of the abstract and results was used, and the number of significant between-group comparisons was recorded. Discrepancies were resolved by discussion. The funding sources of the included reports were not charted.

### 2.8. Risk-of-Bias Assessment of the Direct Comparisons

The risk of bias of the 21 direct comparisons was assessed for their primary or, where none was declared, principal EARR outcome, with RoB 2 for the three randomised trials [31] and ROBINS-I (2016 version) for the 18 non-randomised studies, including the one described as randomised only in its abstract [32]. Case complexity, extraction, the type of planned incisor movement and age were specified before the assessment as the important confounding domains; treatment duration was considered a consequence of the appliance rather than a confounder. Two reviewers (B.L.(E.)G., E.(P.)R.) judged each study independently and resolved disagreements by discussion. The judgements were used to interpret the direct comparisons and not to exclude studies. The assessment was added during peer review (October 2026); ROBINS-I was used in its 2016 version because version 2 was still a draft at the time.

### 2.9. Synthesis of Results

Findings were summarised descriptively and in tables, stratified by review question, treatment phase (comprehensive treatment or a single movement phase) and imaging modality, following JBI recommendations for the presentation of results in scoping reviews [33]. Arithmetic between-group differences are reported to describe the range of values but were not pooled. For each direct comparison, the direction of effect on the primary or principal EARR outcome is reported as concluded in the source report, with the number of significant between-group comparisons, so that results are not counted across multiple teeth, roots or metrics; findings supported by a single study are identified as such. Reporting the direction of effect of each comparison is a descriptive form of synthesis (vote counting based on the direction of effect): it does not estimate the size or precision of an effect, and the counts are interpreted together with study design, risk of bias and measurement error (Section 4.1 and Section 4.2). No clinical recommendation on appliance selection is derived from the mapped evidence.

### 2.10. Use of Generative Artificial Intelligence

During the preparation of this manuscript, the authors used Claude (Opus 5.5; Anthropic, San Francisco, CA, USA) for language editing, for drafting and revising text and for preparing the graphical abstract from the results reported in the manuscript, and DeepL Translator (web version, https://www.deepl.com; DeepL SE, Cologne, Germany) for language refinement. The tools were not used to make selection, data-consistency or risk-of-bias decisions; these decisions and the interpretation of the findings were made by the authors.

## 3. Results

### 3.1. Selection of Sources of Evidence

**Figure 1:**
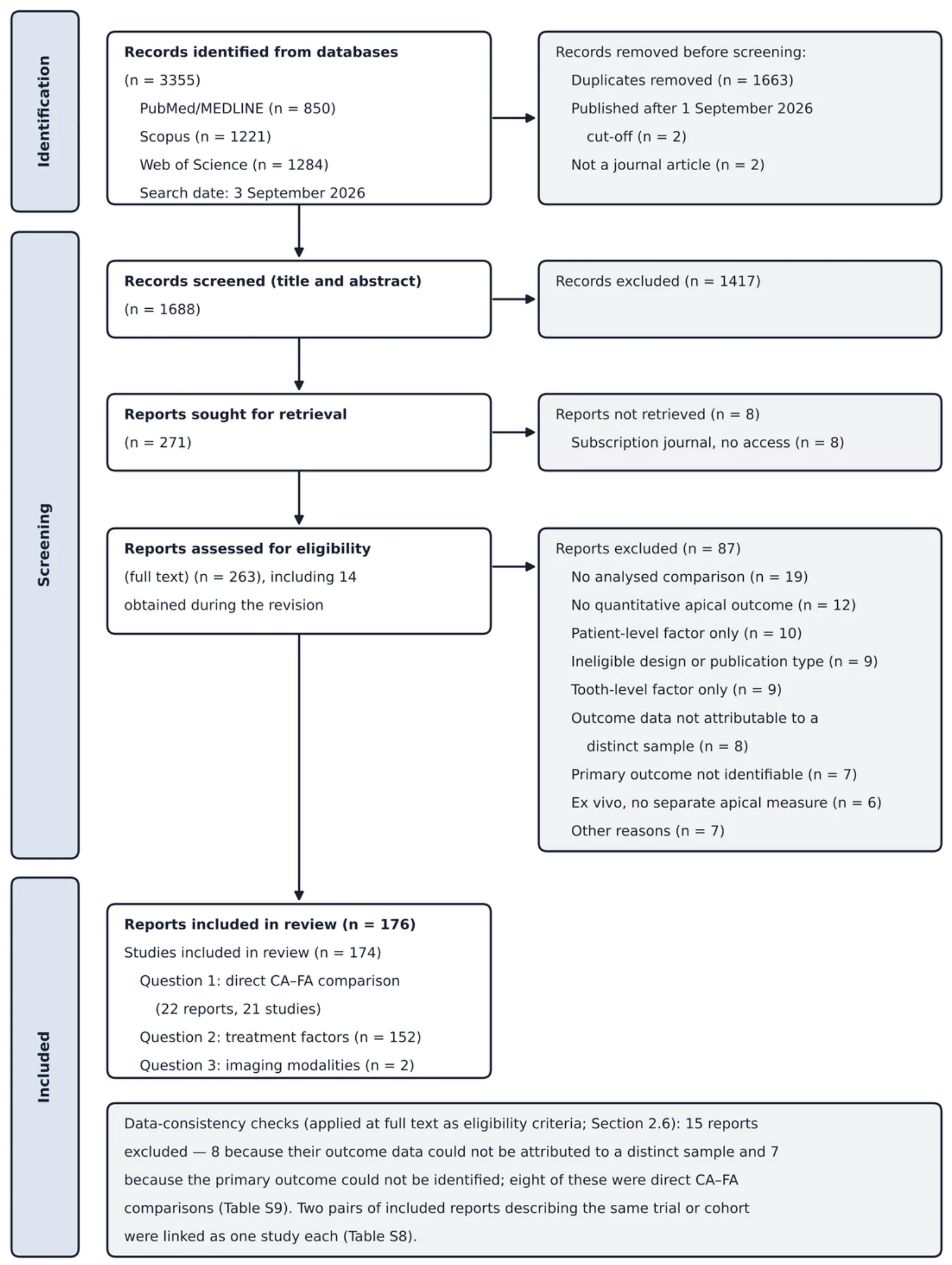
PRISMA-ScR flow diagram of the selection of sources of evidence. CAs, clear aligners; FAs, fixed appliances.

The searches identified 3355 records. After removal of 1663 duplicates and of four records for other reasons (two published after the 1 September 2026 cut-off and two not corresponding to journal articles), 1688 records were screened on title and abstract and 1417 were excluded. Of the 271 reports sought for retrieval, 263 were obtained, 14 of them during the revision (Section 2.5), and eight (3.0%) could not be retrieved (Table S5), three of them published in the *European Journal of Orthodontics*. The 263 reports were assessed for eligibility; 87 were excluded (Table S7) due to no analysed comparison or association (*n* = 19), no quantitative imaging-based apical outcome (*n* = 12), patient-level factor only (*n* = 10), ineligible design or publication type (*n* = 9), tooth-level factor only (*n* = 9), outcome data not attributable to a distinct sample (*n* = 8), primary outcome not identifiable (*n* = 7), ex vivo without a separate apical measure (*n* = 6), and expansion appliance without an apical measure (*n* = 2), with one report each for variants of the same imaging technique, ineligible population, non-orthodontic or nonapical resorption, resorption attributed to osteotomy and non-empirical data excluded. In total, 176 reports of 174 studies were included; two pairs of reports described the same trial or cohort and were linked (Table S8). The selection process is shown in Figure 1.

### 3.2. Characteristics of the Included Reports

The 176 included reports were published between 2020 and 2026 (2020, *n* = 16; 2021, 23; 2022, 19; 2023, 28; 2024, 27; 2025, 35; 2026, 28) and are characterised individually in Table S10. Twenty-two reports addressed Question 1 (five of them also analysed a treatment-related factor), 152 Question 2 and two Question 3. Where the design was stated, 81 reports were randomised trials (29 with a split-mouth design), 69 retrospective cohorts, 13 prospective non-randomised studies and two case–control studies; the design was not stated in 11 reports, one of which was described as randomised in its abstract only. CBCT was used in 106 reports (together with three-dimensional surface or dental models in four), panoramic radiography in 46, periapical radiography in 26 and cephalograms in 10; one report used two-dimensional radiographs and one computed tomography without specifying the type for the measurements, two used ex vivo micro-CT or profilometry, and 18 used more than one modality. Based on the country of the first author’s affiliation, the most frequent were India (33 reports), China (27), Türkiye (19), Egypt (15), Syria (10), Saudi Arabia (8) and the United States (8); the affiliation country could not be established in seven reports.

### 3.3. Direct Comparisons Between Clear Aligners and Fixed Appliances

Twenty-two reports of 21 studies compared EARR directly between CAs and FAs (Table 2). Four studies described their allocation as random. Two reports described the same trial, registered after recruitment had begun, with concealed allocation, at six months [34] and at the end of treatment [35]. A second trial with concealed allocation, also registered after enrolment had begun, compared aligners with fixed appliances and miniscrews during six months of maxillary molar intrusion, so that appliance and force system differed together [16]. One study allocated patients by coin toss, without a described method of allocation concealment [36], and one study of 93 patients stated only in its abstract that patients were “randomly divided”, whereas its methods describe a recruited cohort [37]; a previous meta-analysis classified this study as a retrospective cohort [8]. The remaining studies comprised one prospective comparative study with unreported allocation [38] and 16 retrospective cohorts [12,39,40,41,42,43,44,45,46,47,48,49,50,51,52,53]. One of these reported its comparison on the 11-patient-per-group test set of an artificial-intelligence segmentation study [44]; one, the largest (180 patients), compared appliances as a subgroup analysis and as a covariate within a study of incisor retraction distance [50], and one analysed appliance type as one of several factors in a split-mouth comparison of root-filled and vital teeth [52]. Six studies were restricted to, or reported subgroups of, premolar-extraction treatment [41,46,48,49,50,53], one compared appliances in the orthodontic–orthognathic treatment of skeletal Class III malocclusion [47] (a later report from the same centre [54] was excluded; Section 3.6), and one hospital cohort compared appliances at patient level on panoramic radiographs [51]. Three studies assessed only the first six months of treatment [16,34,36]; the others assessed comprehensive treatment. Analysed sample sizes ranged from 10 to 137 patients per group.

Imaging was CBCT in 15 reports [12,16,35,37,40,41,42,43,45,46,47,48,49,52,53], periapical radiography in three [34,36,38] and panoramic radiography in four [39,44,50,51]. Where reported, CBCT voxel size was 0.125–0.400 mm; one report gives two voxel sizes for the same protocol [46]. Measurement error was expressed in millimetres in three studies only—a Dahlberg error of 0.02–0.03 mm [16], Bland–Altman limits of agreement of −0.60 to 0.50 mm for surface distances [35] and a repeatability standard deviation of less than 0.86 mm [50]; the others reported intraclass correlation or kappa coefficients, which do not express error in millimetres, or no reliability statistic [37,38,42,51,52,53]. EARR metrics ranged from linear incisal edge–apex, cusp tip–apex or CEJ–apex distances, with or without crown-ratio correction, to proportional length reduction, root–crown ratios, three-dimensional surface or apex distances and volumes; five studies reported volumetric loss in addition to linear values [42,43,45,48,53], and in two of them the significance of the between-appliance difference depended on the metric [43,45]; in a third, the volumetric difference concerned only fixed appliances combined with miniscrews [53]. Teeth or roots were the unit of analysis in most studies; one study averaged the two sides of each patient [16], one analysed one pair of teeth per patient [52], one reported a patient-level analysis alongside the tooth-level analysis [44], one analysed patients only, as the presence and grade of resorption in any tooth [51], and one, the largest, used generalised estimating equations to account for the clustering of teeth within patients [50]; no other study did so. Case complexity was controlled by randomisation in three randomised studies [16,34,35,36] and assessed with the American Board of Orthodontics Discrepancy Index, by matching or baseline comparison, in seven retrospective studies [12,39,40,41,45,46,48]; the other studies, including the one described as randomised in its abstract only, relied on eligibility criteria alone [37,38,42,43,44,47,49,50,51,52,53]. The groups differed in treatment duration in six studies—longer with aligners by 7 to 15 months in three [46,49,50] and longer with fixed appliances in three [39,43,51]—in age in three [36,43,51], and in the proportion of extraction cases in one (2.3% with aligners vs. 59.6% with fixed appliances) [51].

Eight studies declared an EARR outcome as primary or based their sample size on it [34,35,39,40,41,46,47,48,50]; in one of them [50] and in two others [49,52], the appliance comparison was secondary or exploratory. The registered trial found no difference between appliances at six months, apart from one of eight teeth, or at the end of treatment (median −0.71 vs. −0.72 mm) [34,35]. The 93-patient study found no significant difference at any of the six anterior tooth positions overall; its subgroup differences by skeletal class went in both directions and were reported without means or standard deviations [37]. The molar-intrusion trial found less root shortening with aligners than with fixed appliances and miniscrews after six months (0.21–0.24 vs. 0.38–0.47 mm per root), with about half the amount of intrusion [16], and the coin-toss randomised study found less EARR with CAs after six months (0.54 vs. 0.99 mm; five of eight teeth) [36]. Of the 17 studies not described as randomised, 15 reported less EARR with CAs on their primary or principal outcome [12,38,39,40,41,42,43,44,45,46,47,48,50,51,52], one did so in only three of eight exploratory root comparisons [49], and one found no difference, with greater volumetric loss only when fixed appliances were combined with miniscrews [53]. The number of between-appliance comparisons per study ranged from one to 36, and in seven studies no more than half of them were significant [34,37,41,45,47,49,53]. Where reported in millimetres, the between-appliance differences on the principal outcome ranged from 0.01 mm [35] to 1.12 mm [46]; in the premolar-extraction studies they ranged from 0.5 to 1.1 mm for individual tooth types [41,46,48], and the orthodontic–orthognathic study reported 0.24 mm [47]. One study reported larger differences in root length (1.8–4.4 mm with fixed appliances against 0.2–0.5 mm with aligners), whereas its volumetric loss differed only from the aligner group treated with bite ramps and its groups differed by 15 years in mean age [43]; in another, length change did not differ between appliances (*p* = 0.976) whereas volumetric loss did [45]. Two studies reported other metrics: the relative root–crown ratio was 4.27 percentage points higher with aligners after adjustment for clustering [50], and resorption of 3 mm or more affected 4.7% of aligner and 36.8% of fixed-appliance patients (adjusted odds ratio for fixed appliances 5.08, 95% CI 1.13–22.83) [51]. Recorded discrepancies of retained reports are described in Section 3.6.

**Figure 2:**
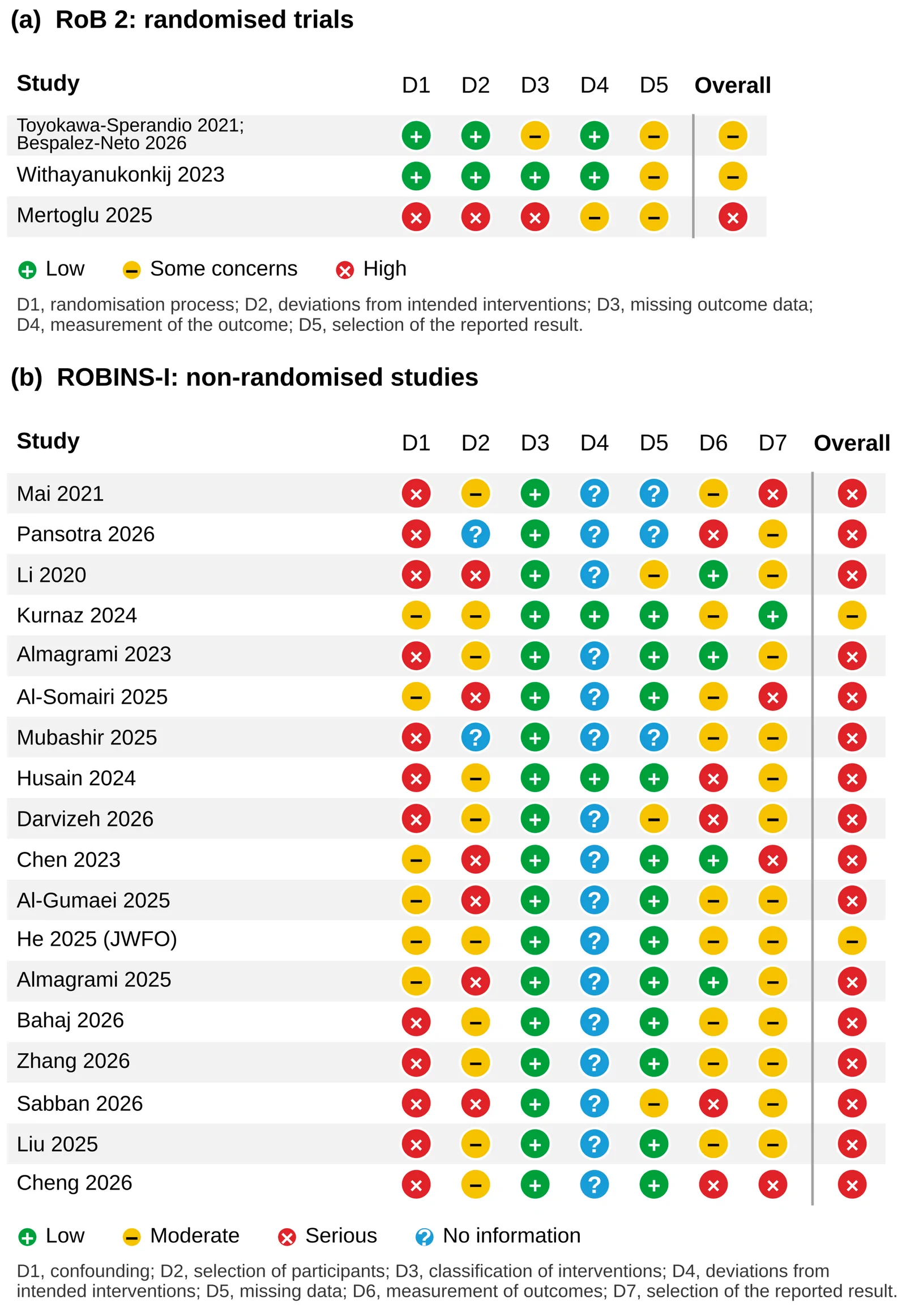
Risk of bias of the direct CA–FA comparisons, judged for the primary or principal EARR outcome: (**a**) RoB 2 for the three randomised trials [16,34,35,36]; (**b**) ROBINS-I for the 18 non-randomised studies [12,37,38,39,40,41,42,43,44,45,46,47,48,49,50,51,52,53]. Reasons for each judgement are given in Table S12. CAs, clear aligners; EARR, external apical root resorption; FAs, fixed appliances.

Risk of bias (Figure 2; Table S12) was judged as of some concern for two of the three randomised trials [16,34,35] and of high concern for the third [36]. Of the 18 non-randomised studies, two were at moderate [39,47] and 16 at serious risk of bias, most often because confounding was not controlled (12 studies) or because participants were selected on characteristics observed after the start of treatment, such as treatment outcome, completion of all aligners or good compliance (five studies).

### 3.4. Treatment Factors Analysed in Relation to EARR

One hundred and fifty-two reports analysed a treatment factor as their primary question (Table S15); five of the direct comparisons also analysed treatment factors—retraction distance, movement pattern and miniscrew anchorage [50], retraction, intrusion and torque within each appliance [46], conventional versus self-ligating brackets [45], extraction and treatment duration [52], and miniscrew anchorage [53]—and are counted under Question 1. As specified in the review question, the Question 2 reports were mapped by factor, design and imaging modality; their findings were not charted. The factors that may confound or mediate the CA–FA comparison—extraction and type of tooth movement, and treatment duration as a possible mediator—are found mainly in four categories: treatment protocols (23 reports), multifactor analyses (23), force magnitude, activation and retraction mechanics (16), and skeletal anchorage and intrusion mechanics (16). The largest group (51 reports) evaluated adjunctive procedures intended to accelerate tooth movement or modulate the biological response, mostly in randomised, often split-mouth, trials in which root resorption was one of several outcomes; these, and the 18 reports on archwires and brackets, relate to the comparison only indirectly. Four reports combined pulpal status with a treatment factor, and one analysed extraction protocols within aligner treatment. Case complexity, a characteristic of the patient, was outside the concept of Question 2 (Table S3, clarification 1); its control in each direct comparison is reported in Table S14. One report of maxillary molar roots in premolar-extraction treatment entered appliance type as a covariate, without values per appliance, and is therefore mapped under Question 2 [55].

### 3.5. Comparisons of Imaging Modalities

Only two reports within the eligibility window compared imaging modalities (Table 3). In 30 patients after en-masse retraction, panoramic radiography classified 39.2% of 120 maxillary incisors as free of resorption against 16.6% on CBCT, with significant differences for all four incisors; the CBCT scans had been taken for other clinical indications and no pre-treatment CBCT existed [13]. In a pilot study of 155 incisors and canines imaged with both modalities within three months, Malmgren scores on panoramic radiographs were higher than on CBCT in the transversal and sagittal planes (mean differences 0.425 and 0.199 grades, *p* < 0.0001) but lower than in the axial plane, where the CBCT score was dichotomised, inter-observer reliability was lower on panoramic radiographs (ICC 0.42 vs. 0.71), and the two-dimensional score produced 28–35% false positives or negatives for the presence of resorption [56]. The two studies thus disagree on the direction of the two-dimensional error—underestimation in one [13] and overestimation in the transversal and sagittal planes in the other [56]—and neither measured linear root loss; the second was one of only two studies among those charted from full texts that modelled the clustering of teeth within patients [56], the other being the largest direct comparison [50]. Two further imaging comparisons were excluded at full text, one because it reported diagnostic confidence rather than a quantitative apical outcome and one because the comparison with micro-CT was made ex vivo without a separate apical measure (Table S7).

### 3.6. Consistency of Reported Data

Of the 263 reports assessed at full text, eight were excluded under criterion (i), because their outcome data could not be attributed to a distinct sample, and seven under criterion (ii), because the primary EARR outcome could not be identified (Table S9); the 15 reports represent 5.7% of those assessed. Of the 32 reports of direct CA–FA comparisons assessed at full text, 22 (21 studies) were included and 10 excluded: eight (25%) under these criteria, one as a report whose tabulated values are stated to be illustrative [57] and one because resorption was graded by visual inspection without defined grades or measurements (Table S7). Nine reports for which criterion (ii) was assessed and not met were retained because their primary outcome could be identified (Table S9a); observations that met neither criterion were also recorded for 18 other retained reports of Questions 1 and 3 (Table S9b).

Under criterion (i), the values reported for the CA group of one report (mean EARR 1.12 ± 1.34 mm; maxillary canine 1.53 ± 1.92 mm; maxillary lateral incisor 1.31 ± 1.33 mm) [58] are identical to those reported for the FA group of an earlier report [12], and several outcome values and methods details of another report [59] are identical or near-identical to those of an earlier report [40]; neither pair of reports states a shared cohort, and both later reports also contain values that could not be reconciled under criterion (ii) (Table S9). Three direct comparisons were excluded under criterion (ii) alone [60,61,62]: in each, values reported in different parts of the report could not be reconciled with each other or with the stated sample sizes (Table S9).

Five reports from one centre [63,64,65,66,67], and an earlier report of the same extraction cohort (Table S8), share authors, examiners, measurement protocol and overlapping recruitment periods, and several of their groups described as independent have identical or near-identical values, with no shared cohort stated (Table S9). Under the first operational rule of Section 2.6, the earliest available report of each cohort was retained for assessment and the later reports, including two direct comparisons [63,66], were excluded under criterion (i); the earliest reports were themselves excluded because they analysed patient-level factors only (Table S8). The same rule led to the exclusion of a later report of a split-mouth cohort reported in two publications (Table S8).

Two reports from another centre on the orthodontic–orthognathic treatment of skeletal Class III malocclusion [47,54] share an ethics approval number, author team and group sizes, and have mutually exclusive inclusion criteria, and three of their 12 cephalometric variables have identical values at all three time points; the earlier report [47] was retained and the later one [54] was excluded under criterion (i). Discrepancies that did not meet the exclusion criteria were recorded in retained reports, among them different values for the same group in different parts of one report [40], *p*-values that cannot be reproduced from the printed data [38,52] and statements in the abstract, text or figure captions that differ from the reported results [37,43,53]; the complete list is given in Table S9. A later report [48] from the same institution as a retained comparison [40], with the same ethics approval number, was retained because its sample (four-premolar extraction) excludes that of [40] (non-extraction) and only one pair of baseline values coincides, in different groups (Table S8).

## 4. Discussion

### 4.1. Summary of Evidence

The direct comparative evidence published since 2020 comprises 21 studies, 16 of them retrospective, that differ in imaging modality, outcome metric, unit of analysis and treatment phase; only one modelled the clustering of teeth within patients [50]. Of the four comparisons described as randomised, only the registered trial with concealed allocation that compared the appliances alone found no difference [34,35]; the other three had design limitations—a force system confined to the fixed-appliance arm [16], allocation concealment not described and an older aligner group [36], and randomisation stated in the abstract only [37] (Section 3.3). Fifteen of the 17 studies not described as randomised reported less EARR with aligners on their primary or principal outcome. In four comparisons, an auxiliary was part of the protocol of one arm only—a palatal miniscrew for intrusion [16] or a transpalatal arch [49] in the fixed-appliance arm, miniscrews in one of two fixed-appliance groups [53] and bite ramps in part of the aligner group [43]—so that these compare treatment protocols rather than appliances under equivalent biomechanics; without them, 15 of the remaining 17 comparisons reported less EARR with aligners (Table S13, scenario iv). Non-randomised comparisons of appliances are vulnerable to confounding by case selection, because aligners may be preferentially offered for less complex malocclusions, and by extraction; only seven of the 16 retrospective studies used a standardised index of case complexity. Treatment duration differs in kind: it was regarded as a consequence of the appliance rather than a confounder (Section 2.8), so a difference in duration may be part of the contrast being compared rather than a bias. Where aligner treatment lasted 7 to 15 months longer [46,49,50], the lower values reported with aligners were obtained despite longer exposure; where fixed-appliance treatment was longer [39,43,51], and in one study also involved far more extractions (59.6% vs. 2.3%) [51], both differences favour aligners. The comparative evidence thus remains dominated by retrospective cohorts with sub-millimetre differences; its largest comparison is a subgroup analysis within a study of retraction distance [50], and its only orthodontic–orthognathic comparison comes from a centre whose second report was excluded [47,54].

### 4.2. Measurement as a Source of Heterogeneity

The between-appliance differences reported in the comparative literature have to be judged against the measurement error of the methods used to obtain them and against a threshold of clinical relevance. A difference between group means can be estimated with a precision finer than the voxel size, because averaging reduces random error, so voxel size itself is not the relevant reference; the relevant references are the error of repeated measurement and a prespecified minimal important difference. Only three direct comparisons reported error in millimetres, and only the registered trial related its results to a prespecified difference (0.5 mm) [34] (Section 3.3). The between-appliance differences on the principal outcome (0.01–1.12 mm in all but one study [43]) are below the 2 mm that separates grade 2 from grade 3 of the Malmgren index, which reserves grades 3 and 4 for apical loss from 2 mm to one-third of the root length and beyond [56]. Teeth were treated as independent observations in all but five studies; only one retained comparison modelled the clustering of teeth within patients [50], and a second that reported such a model was excluded because the signs of its coefficients are opposite to the direction of effect stated in its text [61]. Because teeth of the same patient are not independent and the appliance is assigned at patient level, these tooth-level analyses are expected to underestimate standard errors and may overstate statistical significance, which would inflate the apparent predominance of significant differences favouring aligners. Counting a study as favourable when any of its many tooth-, root- or metric-level comparisons were significant would likewise overstate consistency (Section 3.3). The two imaging comparisons used ordinal grading indices and disagree on whether panoramic radiography under- or overestimates resorption relative to CBCT [13,56], so the magnitude of two-dimensional error for linear measurements still rests largely on earlier work [14]. Where between-group differences are of the order of the measurement error of the method, neither their magnitude nor their direction can be interpreted with confidence, and pooled estimates that combine two-dimensional and three-dimensional measurements, linear and volumetric metrics, or tooth-level and patient-level analyses should be interpreted with caution.

From a periodontal perspective, the clinically relevant outcome is not the mean apical loss but the proportion of patients or teeth with severe resorption, which affects crown–root ratio and long-term prognosis [1,2]. Only four of the retained direct comparisons reported a severity grading [12,40,50,51], and only one reported the patient-level prevalence of severe resorption by appliance, on panoramic radiographs and without reliability statistics [51]; this outcome, rather than sub-millimetric mean differences, is the one on which interdisciplinary treatment planning depends and the one least represented in the mapped evidence.

### 4.3. Consistency of the Data in the Comparative Evidence Base

The two reports with identical values [12,58] were pooled in six of the syntheses cited here (Table S9c): the review by Singh et al., whose tables print the data of one report under “Yi et al.” and those of the other under “None et al.” [5]; the meta-analysis by Darvizeh et al., who note that the two “included extraction cases but did not provide separate results” [6]; two 2026 CBCT-based meta-analyses [7,9]; an umbrella review [10]; and, for the aligner arms only, a meta-analysis of root resorption with clear aligners [68]. Five of these compared aligners with fixed appliances [5,6,7,9,10], although the aligner values of one report are identical to the fixed-appliance values of the other. We did not re-analyse these meta-analyses, and the effect of these reports on their pooled estimates is unknown. The review by Singh et al. also included a report [40], several of whose values are identical or near-identical to those of a later report [59]. In the present review, the direction of effect was the same under all five selection scenarios of Table S13, including the selection decisions taken before the two consistency checks (scenario v): in each, more than three-quarters of the comparisons reported less EARR with aligners and none reported more, and adding the eight excluded reports added seven comparisons in the same direction and one with no difference (Table S13b), partly by counting identical values twice. These observations do not concern the methods of the previous reviews, which followed established risk-of-bias tools, but the limits of those tools: they assess how a study was designed and conducted, not whether its data are unique or internally consistent.

Three patterns of overlap were recorded: values identical or near-identical to those of a later publication by different authors, with the same acquisition settings and criteria [58,59]; near-identical values for groups described as independent across several publications of one research group, with no shared cohort stated [63,64,65,66,67]; and identical values for part of the baseline data in two reports from one centre with mutually exclusive samples [47,54]. Retaining the earliest available report is conservative in the last two patterns, because shared patients cannot be established from the published record. Discrepancies were also recorded in retained reports; the line between recording and excluding—whether the primary EARR outcome could be identified—is stated in Section 2.6, and every observation is documented in Table S9 so that readers can apply a different threshold. The checks themselves are simple and inexpensive: comparing outcome values across included studies, recalculating differences from reported pre- and post-treatment values, checking that category counts sum to group sizes and recording shared cohorts stated in the reports. Checks of this kind are part of the tools developed to assess the trustworthiness of trials [18,29,30], and because the differences at stake in this field are a few tenths of a millimetre, we recommend that future syntheses apply them before pooling.

### 4.4. Comparison with Previous Reviews

Most previous syntheses reported less EARR with aligners; a CBCT-based meta-analysis [8] and an earlier meta-analysis of non-extraction cases, which pooled mostly single-arm studies [4], did not, and the four systematic reviews included in an overview, published up to 2022, were rated as of low or critically low methodological quality [11]. The present review identified 22 reports of 21 direct comparisons published since 2020, including a randomised trial with an end-of-treatment CBCT assessment [35] that was not part of either of the 2026 CBCT-based meta-analyses [7,9], a randomised molar-intrusion trial [16], a randomised comparison that is not described as a trial in its title [36], a 93-patient comparison whose title does not name either appliance [37] and a 180-patient comparison reported within a study of retraction distance [50]. By mapping treatment-factor and imaging-modality studies alongside the direct comparisons, the review places the appliance comparison within the wider body of evidence on which its interpretation depends.

### 4.5. Implications for Research

We propose the minimum reporting set in Table 4; each recommendation addresses a gap observed in this review. No clinical recommendation on appliance selection is made.

### 4.6. Limitations

Eight of the 271 reports sought (3.0%) could not be retrieved, three of them in the *European Journal of Orthodontics*, so the map may under-represent studies published there; judging from their titles and available abstracts, none of the eight compares clear aligners with fixed appliances or imaging modalities. The most important methodological weakness of the review is that the protocol was not registered and that its initial version (Table S3, Part A) was reconstructed during manuscript preparation because no dated copy was retained; its scope was revised before the searches, its clarifications were documented after full-text assessment, and two consistency checks during manuscript preparation changed 18 full-text decisions; all amendments and deviations are reported in Table S3. A protocol finalised and registered before screening would have avoided these revisions (Table S3, Part A). Screening and full-text assessment were performed independently in pairs, but no formal calibration exercise preceded them and no agreement statistic was calculated. The search was limited to three databases (Cochrane CENTRAL was not searched), to English-language full texts and to studies published from 2020 onwards, the strategies were not peer-reviewed, and no citation searching, registry or grey-literature search was performed; the earlier literature on movement vectors and imaging accuracy was therefore outside the scope of Questions 2 and 3. Embase, which contributes the largest number of unique records to systematic searches [69], was not accessible to the team, and although restriction to English-language publications has been shown to have little effect on the conclusions of most syntheses [70], omissions on both counts cannot be excluded. Because earlier and non-English publications were not searched, overlap with previous reports of the same research groups could not be assessed: overlap may be under-detected, and the report retained as the earliest available may not be the first. Residual transcription error in the charted values cannot be excluded. Characteristics of the Question 2 reports (Table S10) were charted at a lower level of detail than those of Questions 1 and 3, and their findings were not charted, so Question 2 is answered as a map of the factors, designs and imaging modalities studied rather than of the associations found. The data-consistency checks relied on the published reports alone and involved judgement about whether a discrepancy prevented the outcome from being established; the observations, but not the judgements, are verifiable from Table S9. Because the data-consistency criteria were applied as eligibility criteria, 15 reports are absent from the map; the direction of effect of those that compared appliances is given in Table S13b. Finally, the risk of bias was assessed only for the direct comparisons, during peer review, and the findings of individual studies are reported without weighting by methodological quality.

## 5. Conclusions

The comparative evidence on EARR with clear aligners and fixed appliances published between 2020 and 2026 consists mainly of non-randomised studies, most at serious risk of bias, that differ in imaging, metric and treatment phase, analyse teeth as independent observations and rarely report measurement error in millimetres. The only randomised trial with concealed allocation that compared the appliances alone found no difference, and the other three comparisons described as randomised had design limitations; the lower values reported with aligners in most non-randomised studies come mainly from retrospective cohorts that did not consistently control case complexity or extraction, and in two studies depend on the metric. Of the reports assessed at full text, 5.7%—and a quarter of the reports of direct appliance comparisons assessed—were excluded because their outcome data could not be attributed to a distinct sample or their primary outcome could not be identified, and two publications reporting the same set of values were pooled in six of the previous evidence syntheses examined in this review. These findings come from a review whose protocol was not registered and whose initial version was reconstructed during manuscript preparation; the direction of the comparative evidence was the same under the selection decisions taken before the post hoc changes (Table S13). Future comparisons of EARR between appliances would benefit more from a declared primary outcome, standardised and fully reported measurement, patient-level reporting of severe resorption and explicit data-consistency checks in evidence syntheses than from further pooling of the existing studies.

## Figures and Tables

**Table 1:** Eligibility criteria.

Element	Included	Excluded
Population	Patients with permanent dentition treated with CAs and/or FAs	Animal and in vitro studies; primary or mixed dentition only
Concept	Quantitative imaging-based assessment of apical root resorption attributable to orthodontic tooth movement, addressing (a) a direct CA vs. FA comparison; (b) an analysed association with movement type, extraction, treatment duration or another treatment factor; or (c) a comparison of imaging modalities	Internal, cervical or replacement resorption only; resorption attributable to impaction or other non-orthodontic causes; no quantifiable EARR outcome; comparisons within one modality (devices, settings, software); patient-level or tooth-level exposures only (Table S3, Part B, clarifications 1 and 3)
Context and sources	Primary clinical studies (randomised and non-randomised trials, cohort, case–control and diagnostic comparison studies) published 1 January 2020–1 September 2026 in English	Reviews; case reports and case series; editorials, letters and commentaries; conference abstracts and conference-supplement communications; reports without empirical clinical measurements; full texts not available in English
Consistency of reported data	Outcome data attributable to a distinct sample, and primary outcome identifiable (Section 2.6)	(i) Outcome data and/or methods text identical or near-identical to those of another publication, without a shared cohort stated in either report; (ii) values reported in different parts of the report that cannot be reconciled, so that the primary EARR outcome cannot be identified

CAs, clear aligners; FAs, fixed appliances; EARR, external apical root resorption. The clarifications of the eligibility criteria (eight adopted on 25 September 2026 and two on 27 September 2026) are listed in Table S3, Part B (Table S3b).

**Table 2:** Direct comparisons of EARR between clear aligners (CAs) and fixed appliances (FAs): design, imaging and reported results.

Study [Ref]	Design	*n* Patients (CA/FA)	Phase; Extraction	Imaging; EARR Metric	Reported EARR, CA vs. FA	Direction (Primary or Principal EARR Outcome; Significant Comparisons)
Toyokawa-Sperandio 2021 [34]	RCT	20/20 randomised; 39 analysed	First 6 months; non-extraction	Periapical; incisal edge–apex (mm)	Between-group differences 0.03–0.35 mm across the eight incisors	No difference (1 of 8 teeth CA < FA)
Bespalez-Neto 2026 [35]	RCT (same trial as [34])	15/17 analysed	Comprehensive; non-extraction	CBCT surface models; 3D surface distance (mm)	Median −0.71 vs. −0.72 mm	No difference (0 of 9 comparisons)
Withayanukonkij 2023 [16]	RCT	20/20 analysed	First 6 months (maxillary molar intrusion); non-extraction	CBCT; cusp tip–apex per root (mm)	0.21–0.24 vs. 0.38–0.47 mm across the three roots	CA < FA (3 of 3 roots); FA arm with palatal miniscrew
Mertoglu 2025 [36]	RCT (coin toss)	27/22 analysed	First 6 months; non-extraction	Periapical; incisal edge centre–apex (mm)	Mean 0.54 vs. 0.99 mm	CA < FA (5 of 8 teeth)
Mai 2021 [37]	“Randomly divided” in the abstract only; recruited cohort in the methods	46/47	Comprehensive; non-extraction	CBCT; CEJ line–apex (mm)	No means or SDs printed (figures only)	No overall difference; subgroups by skeletal class in both directions
Pansotra 2026 [38]	Prospective comparative	20/20	Comprehensive; non-extraction	Periapical; root-length reduction (mm)	Maxillary 0.68 vs. 1.21 mm; mandibular 0.52 vs. 0.94 mm	CA < FA (2 of 2)
Li 2020 [12]	Retrospective cohort	35/35	Comprehensive; extraction 54% vs. 40%	CBCT; incisal edge–apex (mm); Sharpe grade	Severity 0.13 vs. 1.12 mm; prevalence 56.3% vs. 82.1%	CA < FA (14 of 14)
Kurnaz 2024 [39]	Retrospective; root-filled vs. contralateral vital molar	29/37	Comprehensive; non-extraction	Panoramic; CEJ–apex, crown-ratio corrected (mm)	Vital 0.78 vs. 1.21 mm; root-filled 0.28 vs. 0.46 mm	CA < FA (5 of 6 comparisons)
Almagrami 2023 [40]	Retrospective	20/20	Comprehensive; non-extraction	CBCT; CEJ line–apex (mm); Sharpe grade	0.31 vs. 0.62 mm; prevalence 69% vs. 83%	CA < FA
Al-Somairi 2025 [41]	Retrospective	30/30 non-extraction + 30/30 extraction	Comprehensive; extraction and non-extraction	CBCT; CEJ line–apex (mm)	Extraction: U2 0.37 vs. 0.96, U3 0.25 vs. 0.90, L3 0.47 vs. 0.97 mm; other comparisons NS	CA < FA (3 of 12 root comparisons, extraction group)
Mubashir 2025 [42]	Retrospective	30/30	Comprehensive; extraction NR	CBCT; CEJ–apex (mm); volume (mm^3^)	U1 0.62 vs. 1.45 mm; U2 0.55 vs. 1.25 mm	CA < FA
Husain 2024 [43]	Retrospective; three groups	12 and 22 (aligners with and without bite ramps)/26	Comprehensive; non-extraction	CBCT; root length (mm), volume (mm^3^)	UR1 11.97→11.74 and 12.37→11.98 vs. 12.18→9.99 mm; length *p* < 0.001 at all teeth; volume significant in 7 of 24 comparisons	CA < FA for root length (12 of 12); volume 7 of 24
Darvizeh 2026 [44]	Retrospective; AI-segmentation test set	11/11	Comprehensive; extraction NR	Panoramic; proportional root-length reduction (%)	2.81% (AI)/4.98% (expert) vs. 8.95%/10.16%	CA < FA (4 of 4)
Chen 2023 [45]	Retrospective; three groups	18/20 conventional and 21 self-ligating	Comprehensive; non-extraction	CBCT; tooth length (mm), root volume (mm^3^)	Length 0.92 vs. 0.87/0.94 mm (NS); volume loss 23.68 vs. 28.24/28.17 mm^3^	CA < FA for volume (3 of 6); no difference in length
Al-Gumaei 2025 [46]	Retrospective; matched on ABO DI	30/30	Comprehensive; first-premolar extraction	CBCT; incisal edge–apex (mm)	0.43 vs. 1.55 mm	CA < FA (1 of 1)
He 2025 (JWFO) [47]	Retrospective; orthodontic–orthognathic, skeletal Class III	30/30	Comprehensive, including surgery; non-extraction	CBCT; CEJ line–apex (mm)	T0–T2 0.4 vs. 0.64 mm; phases NS	CA < FA (T0–T2 only; 1 of 3 phases)
Almagrami 2025 [48]	Retrospective	40/40	Comprehensive; four first-premolar extraction	CBCT; incisal tip–apex (mm); volume (mm^3^)	0.90 vs. 1.85 mm; volume 19.59 vs. 24.28 mm^3^	CA < FA (6 of 6)
Bahaj 2026 [49]	Retrospective; appliance × sinus–root relationship	40/40	Comprehensive; first-premolar extraction	CBCT; root length per root (mm)	FA greater by 0.30–0.37 mm at 3 roots; other roots NS	Exploratory: CA < FA in 3 of 8 root comparisons
Zhang 2026 [50]	Retrospective; appliance as covariate and subgroup	43/137	Comprehensive; maxillary premolar extraction	Panoramic; relative root–crown ratio (%)	rRCR 96.54% vs. 92.27%	CA < FA (4 of 4)
Sabban 2026 [51]	Retrospective; patient level	43/57	Comprehensive; extraction 2.3% vs. 59.6%	Panoramic; CEJ–apex (mm), graded per patient	EARR > 1 mm in at least one tooth 24/43 vs. 49/57; ≥ 3 mm 2/43 vs. 21/57	CA < FA (5 of 6)
Liu 2025 [52]	Retrospective; root-filled vs. contralateral vital tooth	13/56	Comprehensive; extraction in 36 of 69	CBCT; apical displacement after registration (mm)	Root-filled 0.02 vs. 0.53 mm; vital 0.14 vs. 0.84 mm	CA < FA (2 of 2)
Cheng 2026 [53]	Retrospective; three groups	10/10 and 10 with miniscrews	Comprehensive; four-premolar extraction	CBCT; CEJ–apex (mm); root volume (mm^3^)	Values in figures only	No difference between CA and FA; greater central-incisor volume loss with FA and miniscrews

Values are given as CA vs. FA, as printed in the source reports; standard deviations, *p*-values, device and voxel details, examiners, reliability, unit of analysis, control of case complexity and all reported values are given in Table S14, and the risk of bias of each comparison in Figure 2. 3D, three-dimensional; ABO DI, American Board of Orthodontics Discrepancy Index; AI, artificial intelligence; CBCT, cone-beam computed tomography; CEJ, cemento-enamel junction; EARR, external apical root resorption; JWFO, Journal of the World Federation of Orthodontists; L3, mandibular canine; NR, not reported; NS, not significant; RCT, randomised controlled trial; rRCR, relative root–crown ratio; SD, standard deviation; T0, T2, time points as defined in the source report; U1, U2, U3, maxillary central incisor, lateral incisor and canine; UR1, maxillary right central incisor. Years in the first column are those of the bibliographic records (Table S10); years in the reference list follow the journal volume.

**Table 3:** Studies comparing imaging modalities for the assessment of EARR.

Study [Ref]	Country	Design; *n*	Clinical Context	Modalities Compared	Index/Metric	Examiners; Reliability	Unit; Clustering	Reported Difference Between Modalities	Direction
Alassiry 2022 [13]	Saudi Arabia	Retrospective; 30 patients, 120 maxillary incisors	En-masse retraction after first-premolar extraction (6–8 months), fixed appliances	Panoramic (parameters NR) vs. CBCT (Veraview FX800; 66 kVp, 10 mA; voxel “300”, unit NR), post-retraction only	Levander–Malmgren grades 0–4	Single examiner; intra-examiner ICC > 0.9; Dahlberg value NR	Tooth; chi-square per tooth; clustering not addressed	Grade 0: 39.2% (OPG) vs. 16.6% (CBCT); grade ≥ 1: 60.8% vs. 83.4%; *p* < 0.05 for all four incisors; Table 1 per-tooth counts do not sum to Table 2 marginals	Panoramic underestimates vs. CBCT
Michielsens 2023 [56]	Belgium	Retrospective pilot; 20 patients, 155 incisors and canines	Mixed indications (trauma, endodontic, orthodontic); 2D and 3D within <3 months	Panoramic (VistaPano S) vs. CBCT (Accuitomo/Planmeca), scored in axial, transversal and sagittal planes	Malmgren index 0–4 (axial dichotomised)	Two calibrated observers, scored twice; intra-observer ICC 0.756 (2D) vs. 0.827 (3D-overall); inter-observer 0.423 vs. 0.713	Tooth; linear mixed model with random intercepts for subject, tooth and observer (clustering addressed)	2D scores higher than 3D by 0.425 (transversal) and 0.199 (sagittal), *p* < 0.0001; axial reversed; 2D false positives/negatives 28.0–34.8% at cut-off 0 vs. ≥1	Panoramic overestimates vs. CBCT (Malmgren index)

2D, 3D, two- and three-dimensional; CBCT, cone-beam computed tomography; EARR, external apical root resorption; ICC, intraclass correlation coefficient; NR, not reported; OPG, orthopantomogram.

**Table 4:** Reporting recommendations for clinical studies of orthodontic EARR and the findings of this review from which they derive.

Recommendation	Finding in This Review (Section)
Declare an EARR outcome as primary and a prespecified minimal important difference	8 of 21 direct comparisons declared an EARR outcome as primary or based their sample size on it; 1 related its results to a prespecified difference (0.5 mm) [34] (Section 3.3 and Section 4.2)
Report examiner calibration, blinding and the measurement error in millimetres, from repeated measurements	3 of 21 reported error in millimetres; the others reported reliability coefficients only or no reliability statistic (Section 4.2)
State the unit of analysis and account for clustering of teeth within patients	Teeth treated as independent observations in all but 5; 1 modelled clustering [50] (Section 4.2)
Report the prevalence of severe resorption at patient level	4 reported a severity grading; 1 reported the patient-level prevalence of severe resorption [51] (Section 4.2)
Report the imaging device, voxel size, field of view, landmarks, superimposition or reference method and metric with its unit	Voxel size not reported in 4 of 15 CBCT reports and given as two values in a fifth; significance depended on the metric in 2 studies [43,45] (Table 2 and Table S14; Section 4.2)
Report treatment phase, duration and extraction status per group; control case complexity with a standardised index	Duration differed between groups in 6 studies; 7 of 16 retrospective studies used a standardised complexity index (Section 3.3 and Section 4.1)
Describe the mechanics and auxiliaries used in each arm	In 4 comparisons an auxiliary was part of the protocol of one arm only [16,43,49,53] (Section 4.1)
Report baseline and post-treatment values per group, with category counts	Values that could not be reconciled led to 7 exclusions and were recorded in retained reports (Section 3.6; Table S9)
Declare any overlap of participants with previous publications	8 reports were excluded because their outcome data could not be attributed to a distinct sample (Section 3.6)
In randomised comparisons, describe allocation concealment	Of the 4 comparisons described as randomised, 1 did not describe concealment [36] and 1 was described as randomised only in its abstract [37] (Section 4.1)

CBCT, cone-beam computed tomography; EARR, external apical root resorption. Numbers refer to the 21 direct comparisons unless stated otherwise; section numbers in parentheses.

## Data Availability

All data supporting the findings of this review are provided in the Supplementary Materials: search strategies (Table S2), reports not retrieved (Table S5), full-text exclusions with reasons (Table S7), linked reports (Table S8), observations on the consistency of reported data (Table S9), the characteristics of all included reports (Table S10), risk-of-bias judgements (Table S12), the sensitivity analysis (Table S13), the characteristics and reported results of the direct comparisons (Table S14) and the map of Question 2 (Table S15). Title-and-abstract screening decisions for individual records were not retained.

## Supplementary Materials

The following supporting information can be downloaded at https://www.mdpi.com/article/10.3390/jcm15218219/s1. Table S1: PRISMA-ScR checklist; Table S2: Search strategies for PubMed/MEDLINE, Scopus and Web of Science; Table S3: Review protocol (version 1.0, reconstructed during manuscript preparation) with the record of amendments (versions 1.1–1.4, including the 10 clarifications) and deviations; Table S4: Predefined exclusion reasons; Table S5: Reports not retrieved, with reasons; Table S6: Data-charting form; Table S7: Reports excluded at full text, with primary reason; Table S8: Linked reports; Table S9: Observations on the consistency of reported data, with locators; Table S10: Characteristics of the 176 included reports; Table S11: PRISMA-S checklist; Table S12: Risk-of-bias judgements for the direct comparisons (RoB 2 and ROBINS-I), with reasons; Table S13: Sensitivity analysis of the direction of effect under alternative selection decisions; Table S14: Characteristics and reported results of the direct CA–FA comparisons; Table S15: Treatment factors analysed in relation to EARR (Question 2). The PRISMA-ScR checklist is also provided as non-published material.

## Abbreviations

The following abbreviations are used in this manuscript:

ABO DI	American Board of Orthodontics Discrepancy Index
AI	artificial intelligence
CA	clear aligner
CBCT	cone-beam computed tomography
CEJ	cemento-enamel junction
EARR	external apical root resorption
FA	fixed appliance
ICC	intraclass correlation coefficient
JBI	Joanna Briggs Institute
NR	not reported
PRISMA-ScR	Preferred Reporting Items for Systematic Reviews and Meta-Analyses extension for Scoping Reviews
RCT	randomised controlled trial
rRCR	relative root–crown ratio
