# Supplementary material for: External Apical Root Resorption with Clear Aligners Versus Fixed Appliances: A Scoping Review of Comparative Evidence, Treatment-Related Factors, Imaging Modalities and Consistency of Reported Data
Source: J Clin Med. 2026 Oct 22;15(21):8219. doi: 10.3390/jcm15218219 (PMC13764136; doi:10.3390/jcm15218219)
Supplement: Supplementary file 1 [file jcm-15-08219-s001.zip › Table_S1_PRISMA_ScR_R1.pdf]

Table S1. PRISMA-ScR checklist (Tricco et al., 2018) with the location of each item in the manuscript.

Section	Item	No.	PRISMA-ScR checklist item	How the item is addressed	Location
TITLE	Title	1	Identify the report as a scoping review.	Title ("A Scoping Review of …")	p. 1
ABSTRACT	Structured summary	2	Provide a structured summary that includes (as applicable): background, objectives, eligibility criteria, sources of evidence, charting methods, results, and conclusions that relate to the review questions and objectives.	Unstructured abstract (journal format for reviews) covering background, objective, sources of evidence, methods, results and conclusions	p. 1
INTRODUCTION	Rationale	3	Describe the rationale for the review in the context of what is already known. Explain why the review questions/objectives lend themselves to a scoping review approach.	Introduction, paragraphs 2–5	Section 1
	Objectives	4	Provide an explicit statement of the questions and objectives being addressed with reference to their key elements (e.g., population or participants, concepts, and context) or other relevant key elements used to conceptualize the review questions and/or objectives.	Introduction, last paragraph (three review questions); Table 1 (PCC)	Section 1; Section 2.2
METHODS	Protocol and registration	5	Indicate whether a review protocol exists; state if and where it can be accessed (e.g., a Web address); and if available, provide registration information, including the registration number.	Protocol (version 1.0), reconstructed during manuscript preparation because no dated copy was retained, provided as Table S3, Part A; not registered; amendments (versions 1.1–1.4, including the ten clarifications) and deviations recorded in Table S3, Parts B and C	Section 2.1; Table S3
	Eligibility criteria	6	Specify characteristics of the sources of evidence used as eligibility criteria (e.g., years considered, language, and publication status), and provide a rationale.	Table 1 (PCC framework; 1 January 2020–1 September 2026; English; primary clinical studies); 18 exclusion reasons (Table S4); data-consistency criteria	Section 2.2; Section 2.6
	Information sources	7	Describe all information sources in the search (e.g., databases with dates of coverage and contact with authors to identify additional sources), as well as the date the most recent search was executed.	PubMed/MEDLINE, Scopus, Web of Science Core Collection; searched 3 September 2026; no additional sources; no contact with authors	Section 2.3
	Search	8	Present the full electronic search strategy for at least 1 database, including any limits used, such that it could be repeated.	Full strategies for the three databases, with the date limits as run, in Table S2	Section 2.3; Table S2
	Selection of sources of evidence	9	State the process for selecting sources of evidence (i.e., screening and eligibility) included in the scoping review.	Independent screening by five reviewer pairs at title/abstract and full text; arbitration by an eleventh reviewer; retrieval procedure; data-consistency checks	Sections 2.4–2.6
	Data charting process	10	Describe the methods of charting data from the included sources of evidence (e.g., calibrated forms or forms that have been tested by the team before their use, and whether data charting was done independently or in duplicate) and any processes for obtaining and confirming data from investigators.	Predefined charting form (Table S6); Questions 1 and 3 charted from the full texts and verified against the PDFs by two authors; Question 2 charted at a lower level of detail and verified against the full texts by the reviewer pairs; no contact with investigators	Section 2.7
	Data items	11	List and define all variables for which data were sought and any assumptions and simplifications made.	Items listed in Section 2.7 and in the charting form (Table S6)	Section 2.7; Table S6
	Critical appraisal of individual sources of evidence	12	If done, provide a rationale for conducting a critical appraisal of included sources of evidence; describe the methods used and how this information was used in any data synthesis (if appropriate).	RoB 2 and ROBINS-I for the 21 direct comparisons, to qualify the interpretation of Question 1; not used to exclude studies; data-consistency criteria applied as eligibility criteria, not as appraisal	Sections 2.1, 2.6, 2.8
	Synthesis of results	13	Describe the methods of handling and summarizing the data that were charted.	Descriptive synthesis stratified by question, treatment phase and imaging modality; no pooling; direction of effect reported	Section 2.9

Section	Item	No.	PRISMA-ScR checklist item	How the item is addressed	Location
RESULTS	Selection of sources of evidence	14	Give numbers of sources of evidence screened, assessed for eligibility, and included in the review, with reasons for exclusions at each stage, ideally using a flow diagram.	3355 → 1688 → 271 → 263 assessed (8 not retrieved; 14 of the 263 obtained during the revision) → 176 reports of 174 studies; reasons for the 87 exclusions; Figure 1; Tables S5, S7	Section 3.1; Figure 1
	Characteristics of sources of evidence	15	For each source of evidence, present characteristics for which data were charted and provide the citations.	Table 2 (Question 1), Table S15 (Question 2), Table 3 (Question 3); Table S10 (all 176 reports)	Sections 3.2–3.5; Tables 2 and 3; Tables S10 and S15
	Critical appraisal within sources of evidence	16	If done, present data on critical appraisal of included sources of evidence (see item 12).	Risk of bias per study and domain (Figure 2; Table S12); data-consistency observations in Section 3.6 and Table S9	Sections 3.3, 3.6; Figure 2; Tables S9, S12
	Results of individual sources of evidence	17	For each included source of evidence, present the relevant data that were charted that relate to the review questions and objectives.	Table 2 (reported EARR values and direction per study); Table 3; Table S10; Table S14	Sections 3.3, 3.5
	Synthesis of results	18	Summarize and/or present the charting results as they relate to the review questions and objectives.	Sections 3.3–3.6; Tables 2 and 3; Tables S13–S15	Section 3
DISCUSSION	Summary of evidence	19	Summarize the main results (including an overview of concepts, themes, and types of evidence available), link to the review questions and objectives, and consider the relevance to key groups.	Sections 4.1–4.4	Section 4
	Limitations	20	Discuss the limitations of the scoping review process.	Section 4.6	Section 4.6
	Conclusions	21	Provide a general interpretation of the results with respect to the review questions and objectives, as well as potential implications and/or next steps.	Sections 4.5 and 5	Section 5
FUNDING	Funding	22	Describe sources of funding for the included sources of evidence, as well as sources of funding for the scoping review. Describe the role of the funders of the scoping review.	No external funding for the review (Funding statement); the funding sources of the included reports were not charted (stated in Section 2.7)	Section 2.7; Back matter

PCC, Population–Concept–Context; EARR, external apical root resorption. Items 12 and 16 are optional in PRISMA-ScR. Page numbers will be inserted in the typeset version.
