# Supplementary material for: External Apical Root Resorption with Clear Aligners Versus Fixed Appliances: A Scoping Review of Comparative Evidence, Treatment-Related Factors, Imaging Modalities and Consistency of Reported Data
Source: J Clin Med. 2026 Oct 22;15(21):8219. doi: 10.3390/jcm15218219 (PMC13764136; doi:10.3390/jcm15218219)
Supplement: Supplementary file 1 [file jcm-15-08219-s001.zip › Table_S11_PRISMA-S_checklist_R1.pdf]

Table S11. PRISMA-S checklist (Rethlefsen et al., J. Med. Libr. Assoc. 2021, 109, 174–200).

Section / topic	No.	Checklist item	Reported	Location
Database name	1	Name each individual database searched, stating the platform for each.	PubMed/MEDLINE (NLM PubMed interface); Scopus (Elsevier); Web of Science Core Collection, all editions (Clarivate)	Section 2.3; Table S2
Multi-database searching	2	If databases were searched simultaneously on a single platform, state the name of the platform, listing all of the databases searched.	Not applicable; each database was searched on its own platform	Section 2.3
Study registries	3	List any study registries searched.	None	Section 2.3; Table S3, Part C
Online resources and browsing	4	Describe any online or print source purposefully searched or browsed (e.g., tables of contents, print conference proceedings, web sites), and how this was done.	None	Section 2.3
Citation searching	5	Indicate whether cited references or citing references were examined, and describe any methods used for locating cited/citing references.	Not performed	Section 2.3; Section 4.6
Contacts	6	Indicate whether additional studies or data were sought by contacting authors, experts, manufacturers, or others.	Not performed	Sections 2.3 and 2.5
Other methods	7	Describe any additional information sources or search methods used.	None	Section 2.3
Full search strategies	8	Include the search strategies for each database and information source, copied and pasted exactly as run.	Provided for all three databases as run	Table S2
Limits and restrictions	9	Specify that no limits were used, or describe any limits or restrictions applied to a search (e.g., date or time period, language, study design) and provide justification for their use.	Publication years 2020–2026 in each strategy (PubMed publication date; Scopus PUBYEAR; Web of Science PY); records with an earliest publication date after 1 September 2026 removed before screening; no language, design or article-type limits in the searches (English-language full texts required at eligibility)	Section 2.3; Table S2; Table S3, Part C
Search filters	10	Indicate whether published search filters were used (as originally designed or modified), and if so, cite the filter(s) used.	No published search filters; the PubMed strategy excluded records indexed only to animals (NOT animals[MeSH] NOT humans[MeSH])	Section 2.3; Table S2
Prior work	11	Indicate when search strategies from other literature reviews were adapted or reused for a substantive part or all of the search, citing the previous review(s).	The strategies were developed for this review; no strategy of another review was reused	Table S2
Updates	12	Report the methods used to update the search(es) (e.g., rerunning searches, email alerts).	No update; single search on 3 September 2026	Section 2.3
Dates of searches	13	For each search strategy, provide the date when the last search occurred.	3 September 2026 for all databases	Section 2.3; Table S2
Peer review	14	Describe any search peer review process.	Not performed; the strategies were cross-checked against each other for consistency of terms	Table S2; Table S3, Part C; Section 4.6
Total records	15	Document the total number of records identified from each database and other information sources.	PubMed 850; Scopus 1221; Web of Science 1284; total 3355	Section 2.3; Figure 1
Deduplication	16	Describe the processes and any software used to deduplicate records from multiple database searches and other information sources.	Initial deduplication with the duplicate-detection function of Zotero, without manual review; repeated during the revision on the	Section 2.4; Table S2

Section / topic	No.	Checklist item	Reported	Location
			original exports with a script matching records on DOI, PubMed identifier and normalised title (order: Web of Science, Scopus, PubMed), with manual review of every group of matched duplicates and of all records without a DOI, which identified 44 records matched as duplicates but distinct; 1663 duplicates removed (manuscript Section 2.4; Table S2)	
