# Supplementary material for: External Apical Root Resorption with Clear Aligners Versus Fixed Appliances: A Scoping Review of Comparative Evidence, Treatment-Related Factors, Imaging Modalities and Consistency of Reported Data
Source: J Clin Med. 2026 Oct 22;15(21):8219. doi: 10.3390/jcm15218219 (PMC13764136; doi:10.3390/jcm15218219)
Supplement: Supplementary file 1 [file jcm-15-08219-s001.zip › Table_S12_Risk_of_bias.pdf]

Table S12. Risk-of-bias judgements for the direct CA–FA comparisons (RoB 2 and ROBINS-I), with reasons.

Judgements refer to the primary or, where none was declared, principal EARR outcome of each comparison (last column of Table 2). Two reviewers (B.L.(E.)G., E.(P.)R.) judged each study independently and agreed on the judgements by discussion (Section 2.8). Important confounding domains specified before the assessment include: case complexity, extraction, type of planned incisor movement and age; treatment duration was regarded as a consequence of the appliance, not as a confounder.

(a) RoB 2: randomised trials

Study [reference]	D1	D2	D3	D4	D5	Overall	Reasons for judgements other than low risk
Toyokawa-Sperandio 2021 [34]; Bespalez-Neto 2026 [35]	Low	Low	Some concerns	Low	Some concerns	**Some concerns**	D3: 20% of participants missing at the end of treatment, unequal between groups (25% CA vs 15% FA); no sensitivity analysis.<br>D5: registered after recruitment; the end-of-treatment outcome was measured on CBCT surface models, whereas the registry specifies periapical radiographs.
Withayanukonkij 2023 [16]	Low	Low	Low	Low	Some concerns	**Some concerns**	D5: registered after enrolment began (TCTR20201218004: first enrolment 1 September 2020; registered 18 December 2020); the registered primary outcome is volumetric root resorption at six months and at the end of treatment, whereas the report gives linear root length at six months only.
Mertoglu 2025 [36]	High	High	High	Some concerns	Some concerns	**High**	D1: allocation by coin toss; concealment not described; baseline imbalance in age (22.6 vs 17.1 years, $p < 0.001$).<br>D2: post-randomisation exclusions for non-adherence or appliance failure; per-protocol analysis; one exclusion criterion applied to the aligner group only.<br>D3: 15.5% excluded, unequal between groups (24% vs 7%), for reasons related to treatment.<br>D4: outcome measured by the same researcher; blinding not described.<br>D5: no declared primary outcome; registration not shown to be prospective.

RoB 2 domains: D1, randomisation process; D2, deviations from intended interventions (effect of assignment); D3, missing outcome data; D4, measurement of the outcome; D5, selection of the reported result. Overall: low if all domains are at low risk; high if any domain is at high risk or if some concerns in several domains substantially lower confidence in the result; otherwise some concerns [31].

(b) ROBINS-I: non-randomised studies

Study [reference]	D1	D2	D3	D4	D5	D6	D7	Overall	Reasons for judgements other than low risk
Mai 2021 [37]	Serious	Moderate	Low	No info	No info	Moderate	Serious	**Serious**	D1: allocation not described (randomisation stated in the abstract only); no complexity index; planned movement NR; unadjusted.<br>D2: entry conditional on a usable post-treatment CBCT.<br>D6: single examiner; blinding and reliability NR.<br>D7: results in graphs only; the abstract reports only the significant subgroups.
Pansotra 2026 [38]	Serious	No info	Low	No info	No info	Serious	Moderate	**Serious**	D1: basis of allocation and baseline characteristics NR; unadjusted.<br>D2: recruitment NR. D5: number analysed NR.<br>D6: periapical technique, software, examiners, blinding and reliability NR.<br>D7: primary outcome not declared; statements without data.
Li 2020 [12]	Serious	Serious	Low	No info	Moderate	Low	Moderate	**Serious**	D1: extraction 54% vs 40%; 4 of 10 ABO DI components differ; unadjusted.<br>D2: selection based on post-treatment “treatment outcome quality”.<br>D5: 742 of 840 teeth analysed; reasons NR.<br>D7: no protocol; many tooth-level comparisons.
Kurnaz 2024 [39]	Moderate	Moderate	Low	Low	Low	Moderate	Low	**Moderate**	D1: complexity, extraction, movement and age controlled by restriction or ANCOVA; residual confounding possible.<br>D2: completers only.<br>D6: panoramic radiographs; blinding NR.
Almagrami 2023 [40]	Serious	Moderate	Low	No info	Low	Low	Moderate	**Serious**	D1: incisor movement differed between groups (change in U1–PP, $p = 0.030$); unadjusted.<br>D2: groups matched on ABO DI and pre-treatment measures; number screened NR.<br>D7: no registration; FA mean printed with three different values.

Study [reference]	D1	D2	D3	D4	D5	D6	D7	Overall	Reasons for judgements other than low risk
Al-Somairi 2025 [41]	Moderate	Serious	Low	No info	Low	Moderate	Serious	**Serious**	D1: groups matched on treatment difficulty and comparable in age, duration and ABO DI; restricted to adults with Class I malocclusion; unadjusted.<br>D2: selection criteria included “outcomes” (post-treatment information).<br>D6: outcome assessment not blinded (acknowledged).<br>D7: statements in the abstract and discussion not supported by the printed p-values.
Mubashir 2025 [42]	Serious	No info	Low	No info	No info	Moderate	Moderate	**Serious**	D1: extraction, malocclusion and complexity NR; unadjusted.<br>D2: centre, period and screening NR. D5: number per tooth group NR.<br>D6: examiners, blinding and reliability NR.<br>D7: primary outcome not declared.
Husain 2024 [43]	Serious	Moderate	Low	Low	Low	Serious	Moderate	**Serious**	D1: aligner patients about 15 years older; planned movement differed (intrusion vs proclination); no adjustment for age.<br>D2: entry conditional on CBCT records (selection bias acknowledged).<br>D6: blinding NR; increase in FA root length (UL3 18.7 to 23.2 mm).<br>D7: the abstract emphasises root length.
Darvizeh 2026 [44]	Serious	Moderate	Low	No info	Moderate	Serious	Moderate	**Serious**	D1: no characteristics per group; unadjusted.<br>D2: comparison restricted to an 11-patient test set per group; selection of test cases NR.<br>D5: 22 of 100 included patients used for the comparison.<br>D6: panoramic radiographs; blinding for the reference masks not stated; reproducibility of the AI tool not tested.<br>D7: supplementary tables titled “Synthetic data”.
Chen 2023 [45]	Moderate	Serious	Low	No info	Low	Low	Serious	**Serious**	D1: groups comparable in age, ABO DI, baseline root and movement; unadjusted.<br>D2: aligner patients selected on completion of all aligners and good compliance.<br>D7: no primary outcome; the abstract reports the significant volume result and not the non-significant length result.
Al-Gumaei 2025 [46]	Moderate	Serious	Low	No info	Low	Moderate	Moderate	**Serious**	D1: matched on ABO DI; extraction restricted; differences in torque and intrusion not tested.<br>D2: inclusion required good compliance (post-intervention information).<br>D6: examiners and blinding NR; voxel size given as two values.<br>D7: no registration.
He 2025 (JWFO) [47]	Moderate	Moderate	Low	No info	Low	Moderate	Moderate	**Moderate**	D1: age, durations and crowding NS; restricted to surgical Class III; no complexity index; unadjusted.<br>D2: number screened NR; entry conditional on records at three time points.<br>D6: single investigator; blinding NR.<br>D7: phase changes do not sum to the T0–T2 change; inconsistency between tests.
Almagrami 2025 [48]	Moderate	Serious	Low	No info	Low	Low	Moderate	**Serious**	D1: age, duration, crowding and ABO DI NS; extraction restricted; unadjusted.<br>D2: aligner patients selected on completion of all aligners and refinement; poor compliance excluded.<br>D7: no registration; minor numerical discrepancy.
Bahaj 2026 [49]	Serious	Moderate	Low	No info	Low	Moderate	Moderate	**Serious**	D1: no complexity index; crowding NR; unadjusted.<br>D2: “consecutive” patients but exactly 20 per subgroup; screening NR.<br>D6: blinding NR.<br>D7: appliance comparison exploratory; non-significant p-values not printed.
Zhang 2026 [50]	Serious	Moderate	Low	No info	Low	Moderate	Moderate	**Serious**	D1: no complexity index; regression adjusted for age, sex, duration and post-intervention movement variables.<br>D2: screening NR.<br>D6: panoramic radiographs; blinding NR.<br>D7: appliance is a secondary comparison.

Study [reference]	D1	D2	D3	D4	D5	D6	D7	Overall	Reasons for judgements other than low risk
Sabban 2026 [51]	Serious	Serious	Low	No info	Moderate	Serious	Moderate	**Serious**	D1: large imbalances (extraction 59.6% vs 2.3%, age, sex); multivariable model with 73 events for 7 terms; complexity and movement not controlled.<br>D2: aligner cases partly from external clinicians.<br>D5: row totals differ between outcomes.<br>D6: panoramic radiographs; magnification, reliability and blinding NR.<br>D7: the discussion differs from the results.
Liu 2025 [52]	Serious	Moderate	Low	No info	Low	Moderate	Moderate	**Serious**	D1: characteristics not compared between appliance groups; extraction and duration associated with the outcome; unadjusted.<br>D2: screening NR.<br>D6: single clinician; reliability and blinding NR.<br>D7: appliance a secondary factor.
Cheng 2026 [53]	Serious	Moderate	Low	No info	Low	Serious	Serious	**Serious**	D1: characteristics per group NR; unadjusted.<br>D2: screening NR.<br>D6: device, examiners, reliability and blinding NR; manual segmentation.<br>D7: results in figures only; the abstract differs from the results.

ROBINS-I (2016) domains: D1, confounding; D2, selection of participants into the study; D3, classification of interventions; D4, deviations from intended interventions; D5, missing data; D6, measurement of outcomes; D7, selection of the reported result. Overall: the most severe domain judgement [32]. No info, no information (the report does not state it); reasons are given for the domains judged at moderate or serious risk and, where informative, for those with no information.

Reference numbers refer to the manuscript reference list. CA, clear aligners; FA, fixed appliances; ABO DI, American Board of Orthodontics Discrepancy Index; ANCOVA, analysis of covariance; CBCT, cone-beam computed tomography; AI, artificial intelligence; U1–PP, upper incisor to palatal plane; UL3, upper left canine; NR, not reported; NS, not significant.
