# Supplementary material for: External Apical Root Resorption with Clear Aligners Versus Fixed Appliances: A Scoping Review of Comparative Evidence, Treatment-Related Factors, Imaging Modalities and Consistency of Reported Data
Source: J Clin Med. 2026 Oct 22;15(21):8219. doi: 10.3390/jcm15218219 (PMC13764136; doi:10.3390/jcm15218219)
Supplement: Supplementary file 1 [file jcm-15-08219-s001.zip › Table_S13_Sensitivity_analysis.pdf]

Table S13. Direction of effect on the primary or principal EARR outcome of the direct CA–FA comparisons under alternative selection decisions (descriptive sensitivity analysis).

Direction as defined in Section 2.9 and reported in the last column of Table 2 for the included studies; for the excluded reports, as stated in their abstract and results. The two reports of one trial [34,35] are counted as one comparison.

(a) Scenarios

Scenario	Change from the included set	Comparisons	Less EARR with CA	No difference	Mixed *	More EARR with CA
(i) Included studies (Table 2)	—	21	17 (81%)	3	1	0
(ii) Adding the eight comparisons excluded under the data-consistency criteria (Table S9)	+ [54,58–63,66]	29	24 (83%)	4	1	0
(iii) Removing the five retained comparisons for which a data-consistency criterion was assessed and not met (Table S9(a))	− [40,41,47,48,53]	16	13 (81%)	2	1	0
(iv) Removing the four comparisons in which an auxiliary was part of the protocol of one arm only (Section 4.1)	− [16,43,49,53]	17	15 (88%)	2	0	0
(v) Inclusion decisions before the two consistency checks (Table S3)	− [51,53]; + [54,60,63]	22	19 (86%)	2	1	0

* Less EARR with CAs in 3 of 8 exploratory root comparisons and no difference in the other five [49].

Counts of direction are not a measure of effect: they do not take into account study size, design, risk of bias or measurement error (Section 4.2). Several excluded reports share data with included studies (Table S9), so that scenario (ii) counts some data more than once. In scenario (v), the five reports reassigned from Question 2 to Question 1 by the consistency checks [16,37,43,44,52] are counted as direct comparisons; if they are not counted either, 17 comparisons remain: 15 (88%) report less EARR with CAs, one no difference and one a mixed result, and none more.

(b) Direction reported by the eight excluded comparisons

Report	Reason for exclusion (Table S9)	Primary or principal EARR outcome as reported	Direction
Jyotirmay et al. 2021 [58]	Criteria (i) and (ii)	ARR 1.51 ± 1.34 mm (FA) vs 1.12 ± 1.34 mm (CA); significantly less with CA (abstract)	Less with CA
Gupta et al. 2023 [59]	Criteria (i) and (ii)	0.63 ± 0.21 mm (FA) vs 0.32 ± 0.36 mm (CA), p = 0.000 (abstract)	Less with CA
Su et al. 2026 [60]	Criterion (ii)	Teeth without resorption 52.67% (CA) vs 42.86% (FA), p = 0.010; upper anterior moderate-to-severe resorption 17.14% vs 40.91%, p < 0.001 (abstract)	Less with CA
Issrani et al. 2026 [61]	Criterion (ii)	Root volume loss 6.1 ± 4.8% (CA) vs 8.4 ± 6.1% (FA), p = 0.004; root length reduction smaller with CA, p = 0.001 (Abstract); model coefficients of opposite sign (Table S9)	Less with CA (as stated)
Ay Ünüvar et al. 2025 [62]	Criterion (ii)	No significant difference in tooth-based comparisons, p > 0.05 (abstract)	No difference
Al-Rokhami et al. 2025 [63]	Criterion (i)	1.81 ± 1.55 mm (FA) vs 0.87 ± 0.77 mm (CA), p < 0.001 (abstract)	Less with CA
Al-Rokhami et al. 2026 [66]	Criterion (i)	1.01 ± 0.74 mm (FA) vs 0.32 ± 0.56 mm (CA), p < 0.001 (abstract)	Less with CA
He et al. 2025 (AJODO) [54]	Criterion (i)	Root length reduction T0–T2 1.46 ± 1.12 mm (FA) vs 1.15 ± 1.1 mm (CA), p = 0.029 (Table V; 120 incisors per group); 1.29 ± 0.73 vs 1.09 ± 0.70 mm in the abstract	Less with CA

CA, clear aligners; FA, fixed appliances; ARR, apical root resorption.
