# Supplementary material for: External Apical Root Resorption with Clear Aligners Versus Fixed Appliances: A Scoping Review of Comparative Evidence, Treatment-Related Factors, Imaging Modalities and Consistency of Reported Data
Source: J Clin Med. 2026 Oct 22;15(21):8219. doi: 10.3390/jcm15218219 (PMC13764136; doi:10.3390/jcm15218219)
Supplement: Supplementary file 1 [file jcm-15-08219-s001.zip › Table_S14_Direct_comparisons.pdf]

Table S14. Characteristics and reported results of the direct comparisons of EARR between clear aligners (CAs) and fixed appliances (FAs).

(a) Design, sample, imaging and metric

Study [ref]	Country	Design; allocation	n patients (CA/FA)	Phase; extraction; case type	Teeth (n)	Imaging; device; voxel/FOV	EARR metric; landmarks
Toyokawa-Sperandio 2021 [34]	Brazil	RCT; simple 1:1 randomisation (Excel, external researcher); sealed opaque envelopes; RBR-9zytwf, registered after recruitment began	20/20 randomised; 39 analysed (1 excluded, CA group)	First 6 months; non-extraction; Class I, moderate crowding	Maxillary and mandibular incisors (160/160 at randomisation)	Periapical, paralleling, digital sensor (Schick CDR); 70 kV, 8 mA	Linear, incisal edge–apex (mm)
Bespalez-Neto 2026 [35]	Brazil	RCT (same trial as [34]), end of treatment	15/17 analysed	Comprehensive; non-extraction; Class I, moderate crowding	Incisors (256: 128 lower, 64 U1, 64 U2)	CBCT i-CAT Next Generation, surface models derived from CBCT; voxel 0.3 mm; FOV 17 × 23 cm; kV/mA NR	3D surface point-to-point distance (AI segmentation, SPHARM); maximum, P5, P25 (mm)
Withayanukonkij 2023 [16]	Thailand	RCT; block randomisation, concealed (sealed envelopes); TCTR20201218004, registered after enrolment began; registered primary outcome volumetric root resorption	42 randomised; 20/20 analysed	First 6 months (maxillary molar intrusion); non-extraction; adult anterior open bite	Maxillary first molars, three roots each, sides averaged (20/20 patients)	CBCT Veraviewepocs (Morita); 80 kV, 5 mA; voxel 0.125 mm; FOV 80 × 80 mm; One Volume Viewer	Linear, cusp tip–apex per root (mm)
Mertoglu 2025 [36]	Türkiye	RCT (parallel), coin toss (“randomly divided ... using the coin toss technique”); NCT06709287; concealment and blinding not described	58 allocated; 27/22 analysed (2 CA and 7 FA excluded)	First 6 months (levelling/alignment); non-extraction, no IPR; mild–moderate crowding	Maxillary and mandibular incisors (8 per patient; total NR)	Periapical, paralleling; ImageJ 1.37 with scale calibration	Linear, incisal edge centre–apex (mm)
Mai 2021 [37]	China	“Randomly divided” in the abstract only; methods describe a recruited cohort; method, concealment and blinding NR	93: 46 aligner / 47 self-ligating fixed	Comprehensive (1.85 ± 0.37 vs 1.91 ± 0.40 y, NS); non-extraction; skeletal Class I (15/12), II (19/17), III (12/18)	Six anterior tooth positions, maxillary and mandibular	CBCT, device and voxel NR; Invivo Dental 5	Linear root length, CEJ line–apex (mm); no study-specific measurement error (0.3 mm cited from the literature)
Pansotra 2026 [38]	India (affiliations); setting NR	Prospective comparative; allocation NR	20/20	Comprehensive; non-extraction; mild–moderate crowding	Maxillary and mandibular incisors (n NR)	Periapical; device and parameters NR; software unnamed	Linear root-length reduction (mm); landmarks NR
Li 2020 [12]	China	Retrospective cohort; selected from 578 patients for comparable malocclusion and mechanics	35/35	Comprehensive (21.5 vs 23.3, unit not printed; NS); extraction 54% vs 40%	Maxillary and mandibular anterior teeth; 742 teeth (373 CA / 369 FA)	CBCT 3D Accuitomo; FOV 10 × 10 cm; 85 kV, 4 mA; voxel NR; Dolphin 3D	Linear, incisal edge midpoint–apex (mm); Sharpe grade
Kurnaz 2024 [39]	Türkiye	Retrospective; split-mouth (root-filled vs contralateral vital molar)	29/37	Comprehensive; non-extraction; Class I, moderate crowding	Mandibular molars (29/37 pairs)	Panoramic (Castellini X Radius); parameters NR	Linear CEJ–apex midpoint, crown-ratio corrected (mm); root surface area (mm²)
Almagrami 2023 [40]	China	Retrospective comparative	20/20	Comprehensive (25.9 vs 29.7 months, NS); non-extraction; mild–moderate crowding	Maxillary incisors (160 total)	CBCT KaVo; 120 kV, 5 mA; voxel 0.3 mm; FOV 23 × 17 cm; Invivo 6	Linear, CEJ line–apex (mm); Sharpe grade
Al-Somairi 2025 [41]	China	Retrospective; selected from 500 patients for matched difficulty	30/30 non-extraction + 30/30 extraction	Comprehensive (2.21 vs 2.47 y; 2.95 vs 3.27 y; NS); Class I; extraction (four first premolars) and non-extraction	Incisors (960 total); canines and second premolars (480, extraction groups)	CBCT i-CAT (KaVo 3D eXam); 120 kVp, 37.1 mA; voxel 0.3 mm; FOV 23 × 17 cm; InVivo 6	Linear, CEJ line–apex (mm)
Mubashir 2025 [42]	Saudi Arabia (first author); setting NR (“tertiary orthodontic center”)	Retrospective comparative	30/30	Comprehensive (21.2 vs 20.5 months, NS); extraction NR; mild–moderate crowding	Maxillary incisors, premolars, first molars (n NR)	CBCT, device NR; 90 kV, 10 mA; voxel 0.25 mm; Invivo 5.4	Linear, CEJ–apex (mm); volumetric loss (mm³)
Husain 2024 [43]	USA (IRB University at Buffalo); two private practices, location NR	Retrospective; grouped by treatment received; 1237 records screened (2014–2022)	60: Invisalign with bite ramps 12 / without 22 / FA 26	Comprehensive (1.43 ± 0.53 / 2.01 ± 1.25 / 2.31 ± 0.87 y, p = 0.010); non-extraction; skeletal deep bite, Class I/II, crowding ≤ 6 mm; Damon self-ligating FA	Maxillary canine to canine (six teeth per patient; totals NR)	CBCT i-CAT Next Generation; 120 kV, 5 mA; FOV 17 × 25 cm; voxel NR; Geomagic Control X	Linear root length (mm); root volume (mm³); % volume loss

Study [ref]	Country	Design; allocation	n patients (CA/FA)	Phase; extraction; case type	Teeth (n)	Imaging; device; voxel/FOV	EARR metric; landmarks
Darvizeh 2026 [44]	Spain	Retrospective; AI-segmentation framework with CA–FA comparison	200 radiographs screened (100/100), 100 included (50/50); comparisons on the test set: 11/11 patients (44/44 teeth)	Comprehensive (treatment 2019–2023); extraction NR	Mandibular incisors (44 per group)	Panoramic; single centre; pixel-based measurement (Python)	Proportional root-length reduction (%), incisal edge–apex along tooth axis; AI (U-Net) and expert masks
Chen 2023 [45]	China	Retrospective; three groups (conventional brackets, self-ligating brackets, aligners) selected from records 2016–2020	59: 18 CA / 20 conventional FA / 21 self-ligating FA	Comprehensive (30.94 ± 4.32 vs 28.30 ± 5.08 / 27.67 ± 4.72 months); non-extraction; Class II Division 2	Maxillary central incisors (118; left and right pooled after testing for side differences)	CBCT NewTom 5G or VG; voxel 0.3 mm; kV, mA and FOV NR; Dolphin 11.9, Mimics 17.0	Linear tooth length, incisal tip–apex, midsagittal (mm); root volume (mm³) and % volume loss
Al-Gumaei 2025 [46]	China	Retrospective; groups matched on ABO DI; records October 2016–September 2023	30/30	Comprehensive (43.70 ± 13.36 vs 36.63 ± 10.40 months, p = 0.026); first-premolar extraction; Class I bimaxillary and Class II maxillary protrusion	Maxillary central incisors (60/60)	CBCT 3D Accuitomo; FOV 10 × 10 cm; 85 kV, 4 mAs; voxel given as 0.25 mm and 0.3 mm in the same paragraph; Invivo 6.0.3	Linear, incisal edge midpoint–most apical point (mm)
He 2025 (JWFO) [47]	China	Retrospective cohort; records January 2017–December 2022; orthodontic–orthognathic treatment (bimaxillary surgery)	30/30	Comprehensive incl. presurgical and postsurgical phases (total 42.94 ± 10.57 vs 38.33 ± 9.03 months, p = 0.068); non-extraction; skeletal Class III	Maxillary incisors (120/120)	CBCT KaVo; 120 kV, 5 mA; voxel 0.3 mm; FOV 230 × 170 mm; Dolphin 11.9.20	Linear root length, CEJ reference line–apex, sagittal (mm), at T0, T1 (presurgical) and T2
Almagrami 2025 [48]	China	Retrospective comparative (design term NR); records of one hospital; groups compared on ABO DI (p = 0.858)	40/40	Comprehensive (33.78 ± 5.34 vs 36.20 ± 9.66 months, p = 0.169); four first-premolar extraction; Class II Division 1	Maxillary central and lateral incisors (160/160)	CBCT KaVo; 120 kV, 5 mA; voxel 0.3 mm; FOV 230 × 170 mm; Invivo 6, Mimics 21.0	Linear tooth length, incisal tip–apex, midsagittal (mm) and root volume (mm³), central and lateral incisors separately
Bahaj 2026 [49]	China	Retrospective; four groups of 20 (appliance × maxillary sinus–root relationship type I or V); records 2013–2024; appliance comparison declared exploratory	40/40 (20 per sinus type)	Comprehensive (CA 52.17 ± 7.99 / 48.60 ± 4.61 vs FA 36.85 ± 7.68 / 39.45 ± 9.74 months, p ≤ 0.001); first-premolar extraction; adults	Maxillary second premolar and the three roots of the first molar (root-level analysis; totals NR)	CBCT i-CAT; 120 kV, 18.54 mAs; voxel 0.4 mm; FOV 16 × 13 cm; Invivo 6.0.3	Linear root length from a horizontal reference at the widest crown dimension to the apex (mm), per root
Zhang 2026 [50]	China	Retrospective; 180 adults grouped by incisor retraction distance, with appliance as a covariate and a CA–FA subgroup comparison	180: 43 CA / 137 FA	Comprehensive (49.60 ± 17.50 vs 35.80 ± 9.70 months, p < 0.001); ≥ 2 maxillary premolar extractions; adults	Six maxillary anterior teeth per patient (1080)	Panoramic (Orthoceph OC200D), 400% magnification; lateral cephalograms (Dolphin 11.9); no CBCT	Relative root–crown ratio (rRCR, %; CEJ, apex, incisal edge landmarks); Malmgren index 0–4
Sabban 2026 [51]	Saudi Arabia	Retrospective cohort; hospital records November 2022–September 2025; 249 records screened	100: 43 CA / 57 FA	Comprehensive (20.32 vs 25.51 months, p = 0.005; p = 0.026 in the discussion); extraction in 2.3% vs 59.6% of patients (p < 0.001); simple to moderate Class I–III malocclusion	All teeth with pre- and post-treatment radiographs; outcome recorded per patient (teeth per patient NR)	Panoramic radiographs from the hospital archive; device, parameters and magnification correction NR	Linear root length, CEJ–apex (mm), categorised per patient as none (0), mild (1), moderate (2) or severe (≥ 3 mm); ERR defined as at least one tooth with > 1 mm loss
Liu 2025 [52]	China	Retrospective; split-mouth (root-filled vs contralateral vital tooth); appliance one of several analysed factors	69: 13 CA / 56 FA	Comprehensive (> 1 year; 30.30 ± 10.65 months pooled, per group NR); extraction in 36 of 69 (per group NR)	One root-filled and one contralateral vital tooth per patient (incisors, premolars, molars)	CBCT NewTom VGi; voxel 0.3 mm; FOV 15 × 15 cm; superimposition on the anterior cranial fossa	Linear root-length change (mm): distance between apical points after rigid registration of the pre-treatment tooth on the post-treatment tooth (3D Slicer); root movement measured separately
Cheng 2026 [53]	China	Retrospective; three groups (aligners, fixed appliances, fixed appliances with miniscrews); records 2018–2022	30: 10 CA / 10 FA / 10 FA with miniscrews	Comprehensive (37 ± 11 months pooled, per group NR); four-premolar extraction; skeletal Class II maxillary protrusion	Maxillary incisors (120; 40 per group)	CBCT, device and voxel NR; NNT Viewer 11.5; 3D Slicer 5.7	Linear root length, CEJ centre–apex, midsagittal (mm); root volume (mm³, segmentation)

(b) Examiners, analysis, control of complexity and reported results

Study [ref]	Examiners; reliability	Unit of analysis; clustering	Case-complexity control; baseline differences	Reported EARR, CA vs FA	Direction (primary or principal EARR outcome; significant comparisons)
Toyokawa-Sperandio 2021 [34]	"A single examiner" (methods) but intra- and inter-examiner ICC reported (0.89–0.98; 0.82–0.94)	Tooth; ANCOVA with T0 covariate; clustering not addressed	Randomisation; age, sex, PAR and Little index NS	Between-group differences 0.03–0.35 mm across the eight incisors; only tooth 21 significant (−0.52 ± 0.57 vs −0.86 ± 0.60 mm, p = 0.037); resorption 2.88% of root length overall	No difference (declared primary: EARR per incisor at 6 months; 1 of 8 teeth CA < FA)
Bespalez-Neto 2026 [35]	Intra-examiner ICC 0.808; Bland–Altman bias −0.048 mm, limits of agreement −0.60 to 0.50 mm	Tooth (n = 256); Mann–Whitney, linear regression; clustering not addressed	Randomisation; age (p = 0.099), Little index (p = 0.491), duration 26.2 vs 22.4 months (p = 0.135)	Median −0.71 vs −0.72 mm; all tooth-type comparisons NS (regression p = 0.693)	No difference (declared primary; 0 of 9 comparisons)
Withayanukonkij 2023 [16]	Single examiner; ICC 0.92; Dahlberg 0.02–0.03 mm	Patient (sides averaged), per root type; independent t; no clustering model needed	Randomisation; baseline dentoskeletal variables NS; mechanics differ (aligner bite-block + squeezing vs 150 g coil spring)	MB 0.21 ± 0.02 vs 0.39 ± 0.06; DB 0.23 ± 0.04 vs 0.38 ± 0.06; Pa 0.24 ± 0.03 vs 0.47 ± 0.16 mm (all p < 0.001) (Table 3)	CA < FA (root resorption, first stated objective; 3 of 3 roots); FA arm with palatal miniscrew
Mertoglu 2025 [36]	Single researcher; intra-examiner ICC > 0.90; Bland–Altman (limits NR)	Tooth; paired and independent t; clustering not addressed	Crowding and cephalometrics NS; age differed (22.6 vs 17.1 y, p < 0.001)	Mean 0.54 vs 0.99 mm; 5 of 8 teeth p < 0.05 (e.g., tooth 12: −0.58 vs −1.07 mm; tooth 32: +0.11 ± 3.83 vs −0.79 ± 0.45 mm) (Table 2)	CA < FA (principal outcome, not declared; 5 of 8 teeth)
Mai 2021 [37]	Single physician, three measurements averaged; no reliability statistic; blinding NR	Tooth position; t-tests and ANOVA; clustering not addressed	Age, sex and duration NS; no complexity index; subgroup comparisons by skeletal class (stated objective; no protocol; Class III 12 vs 18)	Overall: no significant difference at any of the six positions (p > 0.05; Figure 4); Class I: fixed higher at all positions; Class II: aligner higher at maxillary central incisors and canines, lower at mandibular canines; Class III: aligner higher at all positions (p < 0.05 in text); no means or SDs printed (Figures 2–7 only); captions of Figures 5 and 7 state p > 0.05 and mark p < 0.05	No overall difference (principal outcome, not declared); subgroups: 7 of 24 comparisons CA < FA, 8 CA > FA (text)
Pansotra 2026 [38]	NR	NR; independent t; clustering NR	Eligibility criteria only	Maxillary 0.68 ± 0.31 vs 1.21 ± 0.42 mm (p = 0.003); mandibular 0.52 ± 0.27 vs 0.94 ± 0.36 mm (p = 0.01) (Tables 1–2)	CA < FA (principal outcome, not declared; 2 of 2)
Li 2020 [12]	Two blinded examiners; ICC 0.97–0.98	Root/tooth; "paired t test" for independent groups, chi-square; clustering not addressed	ABO DI 18.80 vs 17.14 (p = 0.445); 4 of 10 components differed	Severity 0.13 ± 0.47 vs 1.12 ± 1.34 mm; prevalence 56.3% vs 82.1% (p < 0.001)	CA < FA (prevalence and severity; 14 of 14)
Kurnaz 2024 [39]	ICC intra 0.946, inter 0.936; blinding NR	Tooth; ANCOVA (initial length as covariate); clustering NR	ABO DI NS (11.93 vs 13.41); duration longer with FA (1.96 vs 1.28 y, p < 0.05)	Vital 0.78 ± 0.59 vs 1.21 ± 0.86 mm; root-filled 0.28 ± 0.41 vs 0.46 ± 0.32 mm (ANCOVA p < 0.01); root-surface change also smaller with CA (p < 0.01); proportional resorption NS	CA < FA (declared hypothesis on appliance; 5 of 6 comparisons)
Almagrami 2023 [40]	Examiners blinded (number NR); ICC 0.97 intra, 0.92 inter	Tooth; t / Mann–Whitney, chi-square; clustering not addressed	ABO DI 12.69 vs 14.38 (p = 0.610); baseline NS	0.31 ± 0.42 vs 0.62 ± 0.78 mm (results); FA value 0.68 ± 0.97 in abstract and 0.62 ± 0.54 in discussion; prevalence 69% vs 83% (results) and 68.5% vs 82.5% (discussion)	CA < FA (co-primary aim; sample size based on EARR)
Al-Somairi 2025 [41]	Number NR; not blinded (stated limitation); ICC in supplement (20% re-measured)	Tooth type; independent t; clustering not addressed	ABO DI; treatment duration matched; baseline NS	Extraction: U2 0.37 vs 0.96 (p = 0.002), U3 0.25 vs 0.90 (p < 0.001), L3 0.47 vs 0.97 mm (p = 0.003); other extraction and all non-extraction comparisons NS (Tables 2–3); the abstract names U2 and L1 and the discussion U1, U2, L1 and L2 (non-extraction group)	CA < FA (sample size based on EARR; 3 of 12 root comparisons, all in the extraction group)
Mubashir 2025 [42]	NR	NR; paired and independent t; clustering NR	Eligibility criteria only; age, sex, duration "comparable"	U1 0.62 ± 0.21 vs 1.45 ± 0.32 mm; U2 0.55 ± 0.19 vs 1.25 ± 0.28 mm (p < 0.001); volumetric U1 2.48 ± 0.92 vs 6.35 ± 1.24 mm^3	CA < FA (principal outcome, not declared)

Study [ref]	Examiners; reliability	Unit of analysis; clustering	Case-complexity control; baseline differences	Reported EARR, CA vs FA	Direction (primary or principal EARR outcome; significant comparisons)
Husain 2024 [43]	One investigator re-measured 10 subjects; ICC > 0.9; blinding NR	Tooth; T1–T2 change by linear mixed-effects model per tooth; between-group Kruskal–Wallis with Dunn/Benjamini–Hochberg; clustering across teeth not addressed	No index; age differed (FA 23.2, INBR 39.1 and IBR 38.2 y, $p < 0.001$); duration differed	Root length T1→T2 (Table 3): FA 12.18→9.99 (UR1), 23.19→18.75 (UR3) mm vs Invisalign 11.97→11.74 and 23.37→22.94 (INBR), 12.37→11.98 and 24.74→24.36 (IBR); FA vs both Invisalign groups $p < 0.001$ for all teeth; volume and % volume loss: FA vs IBR significant at 4 and 3 of 6 teeth, FA vs INBR NS (Table 4; the text reports no difference); FA UL3 root length printed as 18.73 → 23.22 mm	CA < FA for root length (12 of 12); volume metrics 7 of 24 comparisons CA < FA
Darvizeh 2026 [44]	Two clinicians drew the masks independently, then by consensus; blinding stated for image selection only (κ 0.84); reproducibility of AI not tested	Tooth (44) and patient (11) analyses; Wilcoxon, Mann–Whitney	Eligibility criteria only; age 13–45 y; no complexity index	Reduction 2.81% (AI) / 4.98% (expert) vs 8.95% / 10.16%; $p < 0.0001$ tooth-level, $p = 0.0025$ / 0.0137 patient-level	CA < FA (principal outcome, not declared; 4 of 4)
Chen 2023 [45]	Two examiners, consultation of a "gold standard" examiner; repeated after 2 weeks; Cohen's κ 0.74–0.95; patient records sealed; blinding otherwise NR	Tooth (118 incisors of 59 patients); one-way ANOVA with post hoc tests (α = 0.025); clustering not addressed	ABO DI 12.00 ± 3.38 vs 13.70 ± 2.99 / 13.48 ± 3.12 ($p = 0.211$; lower CA value noted by the authors as a limitation); age NS; duration $p = 0.087$	Length change 0.92 ± 0.87 vs 0.87 ± 1.08 / 0.94 ± 1.41 mm (ANOVA $p = 0.976$); volume loss 23.68 ± 4.82 vs 28.24 ± 6.44 / 28.17 ± 6.07 mm³ ($p = 0.030$; post hoc $p = 0.019$, 0.016); 8.37 ± 1.67 vs 9.84 ± 2.24 / 10.03 ± 2.05% (ANOVA $p = 0.030$; post hoc $p = 0.028$ vs conventional, above the authors' α of 0.025, and 0.011 vs self-ligating)	CA < FA for volume (principal outcome of the abstract, not declared; 3 of 6 pairwise comparisons); no difference in length
Al-Gumaei 2025 [46]	10% re-measured after 1 month, ICC > 0.95, described as intra-rater in methods and inter-rater in statistical analysis; number of examiners and blinding NR	Tooth (60 per group); t-test, ANOVA, multiple regression; clustering not addressed	ABO DI and baseline items NS (Table 1; stated as "no significant differences (P < 0.05)"); age NS ($p = 0.195$); duration longer with CA ($p = 0.026$)	0.43 ± 0.54 vs 1.55 ± 1.52 mm ($p < 0.001$); within FA, EARR increased with incisive-canal contact or invasion (0.93 / 1.1 / 2.07 mm, $p = 0.025$), not within CA ($p = 0.517$)	CA < FA (sample size based on EARR; 1 of 1)
He 2025 (JWFO) [47]	One investigator, measurements repeated after 2 weeks; ICC 0.8–1; blinding NR	Tooth (120 per group); t-test / Mann–Whitney (methods), one-way ANOVA (Table 5 footnote); clustering not addressed	No complexity index; age ($p = 0.946$), durations and crowding NS; sex 14/16 vs 12/18 (no test); ethics approval number shared with an excluded report [54] (Table S8)	T0–T2 root-length reduction 0.4 ± 0.79 vs 0.64 ± 0.8 mm ($p = 0.020$); T0–T1 0.28 ± 0.83 vs 0.39 ± 0.88 ($p = 0.32$) and T1–T2 0.24 ± 0.75 vs 0.37 ± 0.62 ($p = 0.141$), NS; phase changes do not sum to the T0–T2 value	CA < FA (sample size based on EARR; T0–T2 only, 1 of 3 phases)
Almagrami 2025 [48]	Two examiners (I.A., L.A.A.), 20 patients re-measured, blinded to previous measurements; intra- and inter-observer reliability > 0.9	Tooth (160 per group), by tooth type; t-test / Mann–Whitney; clustering not addressed	ABO DI 6.52 ± 10.85 vs 6.65 ± 11.45 ($p = 0.858$); age ($p = 0.328$) and duration NS	Linear 0.90 ± 0.97 vs 1.85 ± 1.18 mm ($p < 0.001$; central 0.87 ± 0.98 vs 1.86 ± 1.50; lateral 0.88 ± 1.01 vs 1.75 ± 0.94); volume 19.59 ± 8.75 vs 24.28 ± 10.05 mm³ ($p < 0.001$); FA central-incisor length 22.97 → 21.03 (Table III) = 1.94 vs 1.86 in Table IV; linear averages (0.90, 1.85) lie outside or at the limit of the tooth-type values	CA < FA (sample size based on EARR; 6 of 6)
Bahaj 2026 [49]	20 scans re-measured by the same examiner after 2 weeks and by a second examiner; ICC 0.925 (95% CI 0.820–0.970); blinding NR	Root; paired and independent tests on change scores; clustering not addressed	No complexity index; age ($p = 0.473$, 0.310) and sex NS within each sinus type; duration longer with CA in both strata ($p < 0.001$, $p = 0.001$), acknowledged as a confounder and not adjusted for	Type I: FA greater resorption for the second premolar (ΔΔT 0.36 mm, 95% CI 0.09–0.63, $p = 0.010$) and palatal molar root (0.37 mm, 0.12–0.63, $p = 0.006$); type V: distobuccal root (0.30 mm, 0.06–0.55, $p = 0.013$); other roots NS	Appliance comparison exploratory: CA < FA for 3 of 8 root comparisons, others NS
Zhang 2026 [50]	One examiner; 50 patients re-measured after 4 weeks; ICC > 0.75; repeatability SD < 0.86 mm; weighted κ 0.71 for the index; blinding NR	Tooth; generalised estimating equations with exchangeable correlation (clustering of teeth within patients addressed), also for the appliance subgroup comparison; multivariable linear models on patient-averaged data	No complexity index; appliance subgroups: age ($p = 0.115$), sex ($p = 0.478$) and miniscrew use ($p = 0.316$) NS; duration longer with CA ($p < 0.001$); retraction-distance groups: age $p = 0.749$	rRCR 96.54% (95% CI 95.65–97.43) vs 92.27% (91.21–93.33), GEE mean difference −4.27% (−5.65 to −2.89), printed with FA as reference although CA > FA, $p < 0.001$ (Supplementary Table IV); appliance an independent predictor in the multivariable model (β −3.05 to −4.79 per tooth type, $p \leq 0.002$)	CA < FA (declared primary outcome rRCR; appliance a secondary comparison; 4 of 4)

Study [ref]	Examiners; reliability	Unit of analysis; clustering	Case-complexity control; baseline differences	Reported EARR, CA vs FA	Direction (primary or principal EARR outcome; significant comparisons)
Sabban 2026 [51]	Two oral and maxillofacial radiologists; reliability statistic and blinding NR	Patient; chi-square, Fisher and Mann–Whitney tests; binary and ordinal logistic regression	No complexity index; aligner group older (26.80 ± 9.21 vs 19.54 ± 5.76 y, $p < 0.001$; abstract 26.8 ± 19.5) with more females (81.4% vs 50.9%); extraction and duration differed	ERR in 24/43 (55.8%) vs 49/57 printed as 84.5% (49/57 = 86.0%), p = 0.002; ≥ 3 mm ("moderate-to-severe" in results, "severe" in methods) in 2/43 (4.7%) vs 21/57 (36.8%), $p < 0.001$; adjusted OR for FA 5.08 (95% CI 1.13–22.83); ordinal OR 2.83 (0.81–9.92, p = 0.104); no mean values in millimetres	CA < FA (prevalence, principal outcome, not declared; 5 of 6 comparisons)
Liu 2025 [52]	Single clinician; reliability statistic NR; blinding NR	Patient (one tooth pair each); t / Wilcoxon tests; clustering not applicable	No complexity index; baseline differences between appliance groups NR	Root-filled 0.02 ± 0.36 vs 0.53 ± 0.57 mm (p = 0.0011); vital 0.14 ± 0.62 vs 0.84 ± 0.64 mm (p = 0.0019) (Table 1)	CA < FA (appliance a secondary factor; 2 of 2)
Cheng 2026 [53]	Three repeated measurements averaged; number of examiners, reliability and blinding NR	Tooth; t-test / Mann–Whitney; clustering not addressed	Eligibility criteria only; baseline differences between groups NR	Length change: no significant difference among the three groups; central-incisor volume change greater with FA and miniscrews than with CA or FA without miniscrews ($p < 0.0001$); lateral incisors NS; values shown in figures only; the abstract reports no significant between-group differences	No difference between CA and FA (principal outcome, not declared); greater central-incisor volume loss with FA and miniscrews

3D, three-dimensional; ABO DI, American Board of Orthodontics Discrepancy Index; AI, artificial intelligence; AJODO, American Journal of Orthodontics and Dentofacial Orthopedics; ANCOVA, analysis of covariance; ANOVA, analysis of variance; CBCT, cone-beam computed tomography; CEJ, cemento-enamel junction; CI, confidence interval; DB, distobuccal root; EARR, external apical root resorption; ERR, external root resorption; FOV, field of view; GEE, generalised estimating equations; IBR, Invisalign with bite ramps; ICC, intraclass correlation coefficient; INBR, Invisalign without bite ramps; IPR, interproximal reduction; JWFO, Journal of the World Federation of Orthodontists; L1, L2, L3, mandibular central incisor, lateral incisor and canine; MB, mesiobuccal root; NR, not reported; NS, not significant; OR, odds ratio; P5, P25, 5th and 25th percentiles of the surface distances; Pa, palatal root; PAR, Peer Assessment Rating; RCT, randomised controlled trial; rRCR, relative root–crown ratio; SD, standard deviation; SPHARM, spherical harmonics; T0, T1, T2, time points as defined in each source report; U1, U2, U3, maxillary central incisor, lateral incisor and canine; UL3, UR1, UR3, maxillary left canine, right central incisor and right canine; κ, kappa coefficient; ΔΔT, between-group difference in change. Values as printed in the source reports; where a value differs between sections of a report, all versions are given. Years in the first column are those of the bibliographic records (Table S10); years in the reference list follow the journal volume. Reference numbers refer to the manuscript reference list.
