# Supplementary material for: External Apical Root Resorption with Clear Aligners Versus Fixed Appliances: A Scoping Review of Comparative Evidence, Treatment-Related Factors, Imaging Modalities and Consistency of Reported Data
Source: J Clin Med. 2026 Oct 22;15(21):8219. doi: 10.3390/jcm15218219 (PMC13764136; doi:10.3390/jcm15218219)
Supplement: Supplementary file 1 [file jcm-15-08219-s001.zip › Table_S15_Treatment_factors_Question2.pdf]

Table S15. Treatment factors analysed in relation to EARR (Question 2).

Factor category	Reports (n)	Examples	Design	CBCT used
Adjunctive and acceleration procedures	51	Corticotomy, corticision, piezocision, micro-osteoperforation, interseptal bone reduction, PRF/PRP, laser/photobiomodulation, LIPUS, vibration, electrical stimulation	43 RCT / 3 retrospective / 4 prospective / 1 NS	36 of 51
Treatment protocol	23	Extraction vs non-extraction, one vs two phases, fixed functional appliance types, fixed vs removable appliances, tooth- vs bone-borne expansion, surgery-first vs conventional orthognathic sequencing, bite turbos	1 RCT / 15 retrospective / 2 prospective / 5 NS	10 of 23
Multifactor analyses	23	Regression or correlation of patient and treatment factors (duration, extraction, amount of movement), including single-cohort studies (clarification 9)	0 RCT / 18 retrospective / 2 case–control / 3 NS	10 of 23
Archwires, brackets and bracket torque	18	NiTi vs CuNiTi, archwire size and sequence, self-ligating vs conventional, labial vs lingual, torque prescription	14 RCT / 2 retrospective / 1 prospective / 1 NS	9 of 18
Force magnitude, activation and retraction mechanics	16	Coil springs vs chains, T-loop vs Ricketts retractor, activation intervals, en-masse vs two-step retraction, controlled tipping vs bodily movement, inclination change, force levels	11 RCT / 3 retrospective / 2 prospective	13 of 16
Skeletal anchorage and intrusion mechanics	16	Miniscrews vs intrusion arches or archwires, one vs two miniscrews, intrusion magnitude, miniscrews vs extraoral, molar or fixed functional anchorage	8 RCT / 5 retrospective / 3 prospective	10 of 16
Pulpal status combined with a treatment factor	4	Root-filled vs vital teeth with extraction protocol or treatment duration	4 retrospective	0 of 4
Aligner-specific factors	1	Extraction protocols within CA treatment	1 retrospective	1 of 1

CAs, clear aligners; CBCT, cone-beam computed tomography; EARR, external apical root resorption; RCT, randomised controlled trial; PRF, platelet-rich fibrin; PRP, platelet-rich plasma; LIPUS, low-intensity pulsed ultrasound; NiTi, nickel-titanium; CuNiTi, copper-nickel-titanium; NS, design not stated. Design and imaging from Table S10; the five direct comparisons that also analysed a treatment factor are not included.
