# Supplementary material for: External Apical Root Resorption with Clear Aligners Versus Fixed Appliances: A Scoping Review of Comparative Evidence, Treatment-Related Factors, Imaging Modalities and Consistency of Reported Data
Source: J Clin Med. 2026 Oct 22;15(21):8219. doi: 10.3390/jcm15218219 (PMC13764136; doi:10.3390/jcm15218219)
Supplement: Supplementary file 1 [file jcm-15-08219-s001.zip › Table_S2_Search_strategies_R1.pdf]

Table S2. Search strategies.

All three databases were searched on 3 September 2026. Database-specific strategies combined terms for root resorption with terms for orthodontic treatment; no filters for study design, language or article type were applied. Publication-year limits (2020–2026) were written into each strategy with the database's own date field (PubMed: publication date, [dp]; Scopus: PUBYEAR; Web of Science: PY), and the PubMed strategy additionally excluded records indexed only to animals. The publication date of a record was defined as its earliest date of publication (online or print), and records with an earliest publication date after 1 September 2026 were removed before screening.

Database (interface)	Strategy as run	Date limit	Records
PubMed/MEDLINE (NLM)	(("Root Resorption"[MeSH Terms] OR "root resorption"[tiab] OR "EARR"[tiab] OR "OIIRR"[tiab]) AND ("Orthodontic Appliances"[MeSH Terms] OR "Tooth Movement Techniques"[MeSH Terms] OR orthodontic*[tiab])) NOT (animals[MeSH Terms] NOT humans[MeSH Terms]) AND ("2020/01/01"[dp] : "2026/12/31"[dp])	Publication date 2020/01/01–2026/12/31 ([dp], in the string)	850
Scopus (Elsevier)	TITLE-ABS-KEY(("root resorption" OR "EARR" OR "OIIRR" OR "root shortening") AND (orthodontic* OR "tooth movement" OR aligner* OR invisalign OR bracket*)) AND PUBYEAR > 2019 AND PUBYEAR < 2027	PUBYEAR 2020–2026 (in the string)	1221
Web of Science Core Collection, all editions (Clarivate)	TS=(("root resorption" OR EARR OR OIIRR OR "root shortening") AND (orthodontic* OR "tooth movement" OR aligner* OR invisalign OR bracket*)) AND PY=(2020-2026)	PY 2020–2026 (in the string)	1284
Total			3355

Deduplication: Records were imported into Zotero (version 9.0.6; Corporation for Digital Scholarship, Vienna, VA, USA) and duplicates were initially identified with the duplicate-detection function of Zotero, without manual review. During the revision, the deduplication was repeated on the original exports with a script that matched records on DOI, PubMed identifier and normalised title (in the order Web of Science, Scopus, PubMed), with manual review of every group of matched duplicates and of all records without a DOI; this identified 44 records, almost all without a DOI, that had been matched as duplicates but were distinct. In total, 1663 duplicates were removed. Four further records were removed before screening (two published after the cut-off date; two not corresponding to journal articles), leaving 1688 records for screening.

Note on the comparability of the strategies: The Scopus and Web of Science strategies include the free-text terms “root shortening”, “tooth movement”, aligner*, invisalign and bracket*, whereas the PubMed strategy relies on the MeSH headings Root Resorption, Orthodontic Appliances and Tooth Movement Techniques together with the free-text terms root resorption, EARR, OIIRR and orthodontic*. The overlap of the three result sets (1663 duplicates among 3355 records) indicates that the strategies retrieved largely the same literature; records retrieved by only one database were screened like all others. The strategies were not peer-reviewed with the PRESS checklist (Table S3, Part C).
