# Supplementary material for: External Apical Root Resorption with Clear Aligners Versus Fixed Appliances: A Scoping Review of Comparative Evidence, Treatment-Related Factors, Imaging Modalities and Consistency of Reported Data
Source: J Clin Med. 2026 Oct 22;15(21):8219. doi: 10.3390/jcm15218219 (PMC13764136; doi:10.3390/jcm15218219)
Supplement: Supplementary file 1 [file jcm-15-08219-s001.zip › Table_S3_Review_protocol_amendments_R1.pdf]

Table S3. Review protocol and record of amendments and deviations.

Part A presents the initial review plan (version 1.0, undated), reconstructed during manuscript preparation because no dated copy of it was retained. Part B records the amendments adopted during the review, in order (versions 1.1–1.4). Table S3a, at the start of Part B, separates the elements defined before screening from those introduced after full-text assessment and gives the number of decisions affected by each. Part C lists every difference between the protocol and the review as conducted, with the reason and the place in the manuscript where it is reported.

Part A. Protocol, version 1.0 (undated; reconstructed during manuscript preparation)

Measurement and reporting of orthodontically induced external apical root resorption in clinical studies of clear aligner therapy: protocol for a scoping review

Authors: Bianca Luminița (Erhan) Groza, Emilia (Prodea) Rusu, Mariana Păcurar, Maria Popa, Mihai Dan Roman, Adrian-Gheorghe Boicean, Gabriela Bota, Laura Stef, Anca Maria Fratila, Denisa Elena Redean, Sorin Radu Fleacă. Contact: rusu.emilia.24@stud.umfst.ro. Version 1.0 — undated (reconstructed during manuscript preparation).

Section	Text
1. Background and rationale	Clinical studies and two meta-analyses report lower external apical root resorption (EARR) with clear aligners (CAs) than with fixed appliances, but the reported differences are typically a few tenths of a millimetre, comparable to the voxel size of commonly used CBCT protocols and to differences attributable to imaging modality, landmarks, metric definition and analysis at tooth rather than patient level. Comparative groups also frequently differ in treatment complexity and duration. No review has mapped how EARR is measured, analysed and reported in CA research. Because this is a question about the conduct of research rather than the effectiveness of an intervention, a scoping review is the appropriate design (Munn et al., 2018).
2. Review questions	In clinical studies assessing EARR in patients treated with CAs: (1) Which imaging modalities and acquisition parameters (for CBCT: device, voxel size, field of view) are used, and at which time points? (2) Which outcome metrics, reference landmarks, registration methods and definitions of severe EARR are used? (3) How are measurement reliability and examiner blinding assessed and reported? (4) What is the unit of analysis, and is clustering of teeth/roots within patients accounted for? (5) In comparative studies, how are treatment complexity, extraction status, treatment duration and type/amount of tooth movement reported and controlled? Secondary aim: to derive a proposed minimum reporting set for EARR studies in CA therapy.
3. Methodology	JBI methodology for scoping reviews (Peters et al., 2020); Arksey and O'Malley (2005) with Levac et al. (2010); reporting per PRISMA-ScR (Tricco et al., 2018).
4. Eligibility criteria (PCC)	Population: patients of any age with permanent dentition treated with CAs (any system, including in-house), with or without auxiliaries; hybrid CA/fixed treatment only if EARR is reported separately for the CA phase; excluded: primary/mixed dentition only; CAs used only for retention or minor finishing. Concept: any imaging-based assessment of apical root resorption (linear, volumetric, ratio-based or ordinal); excluded: internal, cervical or replacement resorption only; resorption attributable to impacted teeth; simulated/finite-element data. Context/types of sources: primary clinical studies of any design (RCTs, non-randomised trials, cohort, case–control, cross-sectional, case series with ≥ 10 patients), any setting, any language, no date restriction; excluded: reviews, case reports/series < 10 patients, editorials, letters, conference abstracts, protocols without results, and reports without empirical patient data. No exceptions to these criteria will be made during the review; any change will be documented as a protocol amendment with justification.
5. Information sources and search	PubMed/MEDLINE, Embase, Scopus, Web of Science Core Collection, Cochrane CENTRAL (inception to search date); ClinicalTrials.gov and WHO ICTRP; backward citation searching of included studies and relevant reviews; forward citation searching of included studies. No language, date or design filters. The PubMed strategy (Supplementary Material S1 of the protocol) retrieved 107 records in a test run (date not recorded); all strategies will be re-run

Section	Text
	and exported on the same day. The strategy will be peer reviewed with the PRESS checklist by a person not involved in the review.
6. Source selection	Deduplication in Zotero; screening in Rayyan. Pilot of 50 random records by all screeners with discussion of discrepancies. Two reviewers will independently screen titles/abstracts and full texts; disagreements resolved by discussion or a third reviewer. Cohen's κ will be reported for both stages. Full-text exclusions will be listed with reasons. Automation tools will not be used to exclude records.
7. Data charting	Charting form (Supplementary Material S1 of the protocol) piloted on five studies, then applied independently and in duplicate. Items: bibliographic data; design; sample; malocclusion; extraction status; aligner system and auxiliaries; teeth/roots; imaging modality and parameters; timing; EARR metric and formula; landmarks; registration; software; severe-EARR definition; blinding; reliability; unit of analysis and handling of clustering; for comparative studies, allocation method and control of complexity, extraction, duration and movement type/amount; and reported EARR values. Corresponding authors will be contacted once (with one reminder after two weeks) for missing key items.
8. Critical appraisal	Not performed, consistent with the purpose of a scoping review. Completeness of reporting of the charted methodological items is an outcome of the review.
9. Synthesis and presentation	Frequencies and proportions; tables; and an evidence map (modality × metric × design). Reported EARR values will be charted descriptively by measurement approach, without pooling or statistical comparison. The review will not make clinical recommendations regarding appliance choice.
10. Timeline	Searches, screening, charting, synthesis and manuscript: dates left blank in version 1.0.
11. Funding and conflicts of interest	No funding. The authors declare no conflicts of interest, including no relationships with aligner manufacturers.

The protocol's Supplementary Material S1 (aligner-specific search strategies for the sources listed in Section 5 and the planned charting form) is superseded by Table S2 and Table S6 of the manuscript.

Part B. Amendments

Table S3a. Elements of the review defined before screening and elements introduced after full-text assessment

Element	Defined before screening (versions 1.0–1.1)	Introduced after full-text assessment	Decisions affected
Review questions (three)	Yes	—	None
Population–Concept–Context criteria	Yes	—	None
Databases, search strings and publication window	Yes	—	None
Predefined exclusion reasons (18; Table S4)	Yes	—	None
Data-consistency criteria (i) and (ii), general form	Yes	—	None
Screening procedure (five fixed pairs, independent assessment, arbitration)	Yes	—	None
Clarifications 1–8 (version 1.2)	—	Yes	None (record of how borderline reports had been handled)
Operational rules (a) and (b), first consistency check (version 1.3)	—	Yes	3 decisions changed (3 exclusions: R0258, R0501, R0643); 4 reports reassigned from Question 2 to Question 1
Clarifications 9–10, second consistency check (version 1.4)	—	Yes	15 decisions changed (13 inclusions; 2 exclusions: R0259, R0529); 1 report reassigned to Question 1; 5 exclusion codes corrected

Element	Defined before screening (versions 1.0–1.1)	Introduced after full-text assessment	Decisions affected
Risk-of-bias assessment of the direct comparisons (RoB 2, ROBINS-I)	—	Yes	None (not used for selection; Section 2.8)
Total			18 decisions changed (13 inclusions, 5 exclusions): 5 concerned direct CA–FA comparisons (inclusions R0865, R0291; exclusions R0258, R0259, R0529) and 13 concerned Question 2 reports

Version 1.0 was reconstructed during manuscript preparation (Part A); the first column therefore rests on that reconstruction and on version 1.1, which was adopted before any record was retrieved. Each changed decision is listed with its record identifier and the rule applied in the descriptions of versions 1.3 and 1.4 below. Both consistency checks (versions 1.3 and 1.4) were performed independently by D.E.R. and L.S., and each changed decision was confirmed by S.R.F.

Version	Date	Change	Stage of the review at which it was adopted
1.1	Before the searches of 3 September 2026	Scope revised to the three review questions of the manuscript (direct CA–FA comparisons; treatment-related factors; imaging-modality comparisons); population extended to patients treated with fixed appliances; publication window 1 January 2020–1 September 2026; three databases (PubMed/MEDLINE, Scopus, Web of Science Core Collection) with database-specific strategies (Table S2); data-consistency criteria (i) and (ii) added as eligibility criteria at full text; predefined list of 18 exclusion reasons (Table S4); screening by five fixed reviewer pairs with arbitration by an eleventh reviewer; English full texts only.	After the protocol was written and before any search was run; no record had been retrieved or screened.
1.2	25 September 2026	Eight clarifications of the eligibility criteria (Table S3b), recording how borderline reports had been handled at full-text assessment. No selection decision was changed.	After full-text assessment (11–20 September 2026), during data charting.
1.3	26 September 2026	The two operational rules applied under the data-consistency criteria were made explicit (manuscript Section 2.6): (a) when the same research group reports near-identical values for groups described as independent in two or more reports with overlapping recruitment periods and no shared cohort stated, the earliest available report is retained and the later reports are excluded under criterion (i); the same rule applies to reports by different authors that report values identical to those of an earlier publication; (b) criterion (ii) is met only when the primary EARR outcome cannot be identified from the report; reconcilable discrepancies are recorded (Table S9) but do not lead to exclusion. A consistency check of all 246 full-text decisions against these rules changed three decisions—R0258 (Su et al. 2026) from included to excluded under criterion (ii), and R0501 and R0643 (Al-Rokhami et al. 2025, 2026) from included to excluded under criterion (i), with the earliest report R0259 retained—and reassigned four included direct CA–FA comparisons that had been filed under Question 2 to Question 1 (R0596 Withayanukonkij et al. 2023; R1638 Mai et al. 2021; R0296 Husain et al. 2024; R0636 Darvizeh et al. 2026). All counts in the manuscript reflect these decisions.	During manuscript preparation, after data charting.
1.4	27 September 2026	Second consistency check: every full-text decision was checked against the full text of the report, the clarifications of Table S3b and the two operational rules of Section 2.6. (a) Eight reports excluded under R04 or R02 were found to satisfy the eligibility criteria and were included: R0339 (Mohan et al. 2026; archwire type), R0389 (Wagh et al. 2023; micro-osteoperforation vs vibration, ordinal index compared between groups at a common time point, clarification 5), R0473 (Le et al. 2024; coil spring vs elastomeric chain), R0865 (Sabban et al. 2026; direct CA–FA comparison, Question 1), R1257 (Vélez et al. 2025; appliance type), R1401 (Saifeldin et al. 2021) and R1537 (Hasan et al. 2021; low-level laser therapy vs control), and	During manuscript preparation, after data charting.

Version	Date	Change	Stage of the review at which it was adopted
		R1485 (Masaes et al. 2022; T-loop vs Ricketts retractor). (b) Criterion (ii) was applied uniformly with rule (b): five reports previously excluded under R17 whose primary EARR outcome can be identified were included, with their observations recorded in Table S9: R0291 (Cheng et al. 2026; Question 1), R0493 (Pham et al. 2025), R0577 (Abd ElMotaleb et al. 2024), R1297 (Mahmoud et al. 2025) and R1559 (Kaur et al. 2020). (c) Rule (a) was applied to the complete chains of related reports: R0259 (Al-Rokhami et al. 2025, Sci Rep) was excluded under criterion (i), the earliest report of its cohort being R0268 (excluded, R05), and R0529 (He et al. 2025, AJODO) was excluded under criterion (i), R0862 being the earlier report. (d) R0905 (Liu et al. 2025) was reassigned from Question 2 to Question 1 under clarification 10. (e) Five codes were corrected without change of decision: R0153 and R0215 from R05 to R06 (tooth-level factors), R0777 and R1000 from R04 to R12 (protocol articles), R0812 from R04 to R09 (expansion appliance without an apical measure). (f) Clarifications 9 and 10 were added (Table S3b). All counts in the manuscript, Figure 1 and Tables S4, S7–S10 reflect these decisions.	

Table S3b. Clarifications of the eligibility criteria adopted in version 1.2 (25 September 2026; nos. 1–8) and version 1.4 (27 September 2026; nos. 9 and 10)

Concepts (a), (b) and (c) refer to the three components of the Concept criterion in Table 1 of the manuscript: (a) a direct CA vs FA comparison; (b) an analysed association between a treatment-related factor and EARR; (c) a comparison of imaging modalities. Exclusion codes refer to Table S4.

No.	Clarification	Application
1	Reports analysing only patient-level factors (age, sex, skeletal class, overjet, overbite, initial incisor inclination, genetic or systemic factors) do not satisfy concept (b), which requires a treatment-related factor.	R05 (patient-level factor only)
2	Pulpal status (root-filled vs vital teeth) is a tooth-level characteristic and satisfies concept (b) only when analysed in combination with a treatment factor (appliance type, extraction protocol, movement type).	Included under Question 2 when combined (Table S15, "pulpal status combined with a treatment factor"); otherwise R06
3	Reports analysing only tooth-level factors (tooth type, root morphology, root–canal proximity, pre-existing resorption) do not satisfy concept (b).	R06 (tooth-level factor only)
4	Comparisons between variants of the same imaging technique (devices, acquisition settings, software, observers or reconstruction methods) do not satisfy concept (c), which requires different imaging modalities.	R07 (variants of the same technique); reported as within-modality comparisons in Table 1
5	Ordinal resorption indices (Malmgren/Levander, Sharpe, McNab and modifications) are accepted as a quantitative imaging-based outcome when scored from images at two or more time points or compared between treatment groups at a common time point ("rule A"); an index scored on a single post-treatment image without a between-group comparison is not.	Accepted under rule A; otherwise R02 (no quantitative imaging-based apical outcome)
6	Adjunctive and acceleration procedures (corticotomy, piezocision, micro-osteoperforation, platelet concentrates, photobiomodulation, low-intensity pulsed ultrasound, vibration, electrical stimulation) count as "another treatment factor" under concept (b); split-mouth and self-controlled designs (with vs without the procedure in the same patients) satisfy concept (b).	Included under Question 2 (Table S15, "adjunctive and acceleration procedures")
7	Ex vivo analyses of extracted teeth (micro-CT, scanning electron or confocal microscopy) are eligible only when an apical measure (apical shortening or apical-third loss) is reported separately from whole-root surface or crater analysis.	R08 (ex vivo without separately reported apical resorption)
8	Reports of maxillary expansion appliances are eligible only when an apical measure is reported and compared between protocols; orthodontic–surgical reports in which resorption is attributed to the osteotomy are not eligible.	R09 (expansion appliance without apical measure or comparison); R10 (resorption attributed to osteotomy)
9	Single-cohort reports satisfy concept (b) only when EARR is related to at least one treatment-related variable (treatment duration, amount or type of tooth	Included under Question 2 (Table S15, "multifactor analyses"); otherwise R04 (added in version 1.4)

No.	Clarification	Application
	movement, treatment protocol) by between-group comparison, correlation or regression; a pre–post description of a single treatment does not.	
10	Question 1 includes reports that give EARR values separately for a CA group and an FA group treated in the same study, with a between-group comparison as a stated aim or a reported test, whether the appliance is the primary or a secondary factor; reports that enter appliance type only as a covariate, without values per appliance, are assigned to Question 2.	Assignment to Question 1 or 2 (added in version 1.4)

Part C. Deviations from the protocol

Item	Protocol (version 1.0)	Review as conducted	Reason / where reported
Review questions	Five questions on measurement and reporting in CA studies; secondary aim: a minimum reporting set	Three questions: direct CA–FA comparisons; treatment-related factors in CA or FA studies; imaging-modality comparisons	Version 1.1, before the searches. The scope was widened because the appliance comparison cannot be interpreted without the evidence on treatment factors and imaging modalities on which it depends, and because the consistency of the data in the comparative reports had to be checked (Introduction). The five measurement questions of version 1.0 are retained as charting items (Table S6) and inform the minimum reporting set (Section 4.5).
Population	Patients treated with CAs only	Patients treated with CAs and/or FAs	Consequence of the revised questions (version 1.1).
Date restriction	None (inception to search date)	1 January 2020–1 September 2026	Version 1.1. Chosen to map the current comparative evidence; the window begins after the search periods of the earliest meta-analyses of the comparison (manuscript references 3 and 4). Most, but not all, of the studies pooled by the comparative meta-analyses published since 2024 fall within it (Section 2.2). Earlier literature on movement vectors and imaging accuracy is cited from previous work. Reported as a limitation (Section 4.6).
Language	Any language	Full texts in English	Version 1.1; feasibility. Reported as a limitation (Section 4.6).
Information sources	PubMed/MEDLINE, Embase, Scopus, Web of Science Core Collection, Cochrane CENTRAL, ClinicalTrials.gov, WHO ICTRP; backward and forward citation searching	PubMed/MEDLINE, Scopus, Web of Science Core Collection	Version 1.1. Embase requires a subscription that is not included in the institutional subscriptions available to the team. Cochrane CENTRAL, the trial registries and citation searching were not used: the revised questions concern published reports with results of any design, and the review was limited to database-indexed reports. The overlap between the three result sets (1663 duplicates among 3355 records) is reported in Table S2. Reported as a limitation (Section 4.6).
Search strategy	Aligner-specific strategies (protocol Supplementary S1)	Strategies combining root-resorption and orthodontic-treatment terms, with date limits (Table S2)	Consequence of the revised questions (version 1.1); strategies as run are reproduced in Table S2.
Deduplication (repeated check)	Deduplication in Zotero (protocol Section 6)	Initial deduplication with the duplicate-detection function of Zotero, without manual review, as planned. During the revision, the deduplication was repeated on the original exports with a script matching records on DOI, PubMed identifier and normalised title	Counts are reported in Sections 2.4 and 3.1, Figure 1 and Tables S2, S4, S7, S9, S10 and S11.

Item	Protocol (version 1.0)	Review as conducted	Reason / where reported
		(order: Web of Science, Scopus, PubMed), with manual review of every group of matched duplicates and of all records without a DOI; 44 records that had been matched as duplicates but were distinct were identified. Two were removed before screening (one published after the cut-off; one not a journal article); 42 were screened with the same procedure, 39 were excluded on title and abstract and 3 were assessed at full text: 2 were included under Question 2 (R1656, R1688) and 1 was excluded (R07; R1661).	
PRESS peer review of the search	Planned, by a person not involved in the review	Not performed	Not performed. The three strategies were instead cross-checked against each other for consistency of terms (Table S2, note), and the 50% overlap between the result sets was taken as an indication of consistent retrieval. Reported as a limitation (Section 4.6).
Screening platform and pilot	Rayyan; pilot of 50 records by all screeners	Zotero; no formal pilot	Screening was performed in Zotero, which already held the deduplicated records and full texts; no formal pilot was recorded. Disagreements were resolved by arbitration from the first record.
Screening procedure	Two independent reviewers; third reviewer for disagreements; Cohen's κ reported for both stages	Five fixed reviewer pairs, independent screening at both stages; arbitration by an eleventh reviewer; κ not calculated	Version 1.1 (team of eleven authors). An agreement statistic is not required by PRISMA-ScR; its absence is reported as a limitation (Section 4.6).
Automation tools	Not to be used to exclude records	Not used to select or exclude records	Consistent with the protocol; tools used in preparing the manuscript are disclosed in Section 2.10 and in the Acknowledgments.
Data-consistency criteria	Not specified	Two criteria applied as eligibility criteria at full text, with two operational rules (Section 2.6) and the 18 exclusion codes (Table S4)	Added in version 1.1 (before the searches); operational rules made explicit in version 1.3, with three decisions revised, and all full-text decisions re-checked in version 1.4, with fifteen decisions and five codes revised (Part B).
Handling of reports meeting criterion (i) or (ii)	Not specified	Excluded; authors and journals not contacted	The review maps the evidence at a fixed date and does not pool results; all observations are documented in Table S9 so that readers can verify them and classify the reports differently (Section 2.6). This departs from the Cochrane guidance (manuscript reference 17), which provides for seeking clarification from the study authors and, where concerns remain, from the journal editor.
Contact with authors	Once, with one reminder, for missing key items	Not performed for missing items; authors were contacted only to obtain full texts during the revision (next row)	So that every observation on data consistency and every charted value can be verified from the published reports alone (Section 2.6); missing items were charted as “not reported” (Table S6).
Full-text retrieval	Not specified	22 of the 271 reports sought could not be obtained at the initial retrieval; during the revision (October 2026), retrieval was attempted again through an extension of the institutional access agreement, through interlibrary loan and by contacting the	Additional retrieval during peer review, at a reviewer’s request. Reported in Sections 2.5 and 3.1, Figure 1 and Tables S5, S7 and S10.

Item	Protocol (version 1.0)	Review as conducted	Reason / where reported
		corresponding authors; 14 were obtained and assessed at full text with the same procedure (9 included, 5 excluded), and 8 could not be obtained	
Data charting	Independent, in duplicate, piloted on five studies	Questions 1 and 3: all items charted from the full texts and every value verified against the PDF by two authors (B.L.(E.)G., E.(P.)R.); Question 2: design, setting, sample, appliance, analysed factor, imaging modality and metric charted at a lower level of detail and verified against the full text by the reviewer pair that had assessed each report	Feasibility (176 included reports); reported as a limitation (Section 4.6).
Synthesis	Evidence map (modality × metric × design)	Descriptive synthesis stratified by question, treatment phase and imaging modality (Tables 2 and 3; Tables S10, S14 and S15)	Consequence of the revised questions (version 1.1).
Critical appraisal	Not performed	Risk of bias of the 21 direct comparisons assessed with RoB 2 and ROBINS-I; not used to exclude studies	Added during peer review (October 2026), at the reviewers' request, to qualify the interpretation of Question 1 (Section 2.8; Figure 2; Table S12).

CA, clear aligners; FA, fixed appliances; EARR, external apical root resorption; PRESS, Peer Review of Electronic Search Strategies. Section numbers refer to the manuscript.
