# Supplementary material for: External Apical Root Resorption with Clear Aligners Versus Fixed Appliances: A Scoping Review of Comparative Evidence, Treatment-Related Factors, Imaging Modalities and Consistency of Reported Data
Source: J Clin Med. 2026 Oct 22;15(21):8219. doi: 10.3390/jcm15218219 (PMC13764136; doi:10.3390/jcm15218219)
Supplement: Supplementary file 1 [file jcm-15-08219-s001.zip › Table_S6_Data-charting_form_R1.pdf]

Table S6. Data-charting form.

The form was applied to every included report. Items in sections A–K were charted for the reports addressing Questions 1 and 3; for the reports addressing Question 2, items A–C (design, setting, sample), D (analysed factor and appliance), G (imaging modality) and H (metric) were charted and are presented in Table S10. Section L was applied to every report assessed at full text. "NR" (not reported) was entered whenever an item was absent from the report; no value was inferred.

Section	Items charted	Format / permitted values
A. Identification	RecID; first author; year; journal; DOI/PMID; question(s) addressed (Q1/Q2/Q3); linked reports (same trial/cohort)	Free text; Q1/Q2/Q3; RecIDs of linked reports
B. Design and setting	Study design as stated by the authors; allocation method (randomisation, concealment) if any; registration number; country; setting (institution, practice); recruitment or treatment period; ethics approval	RCT (parallel/split-mouth) / prospective non-randomised / retrospective / cross-sectional / other; free text; NR if not reported
C. Participants	Number of patients per group (randomised and analysed); age (mean ± SD, range); sex; malocclusion / case type; extraction or non-extraction; case-complexity index (ABO DI, PAR, Little) and values	n; years; free text; NR
D. Intervention / exposure	Appliance system(s) and brand; bracket slot and prescription; aligner brand and change interval; adjunctive procedure or analysed treatment factor; force magnitude; movement type	Free text; NR
E. Treatment phase and duration	Phase assessed (comprehensive treatment vs single phase: alignment, retraction, intrusion, expansion); time points (T0, T1 … definitions); treatment duration per group (mean ± SD)	Comprehensive / single phase (specify); months or years
F. Teeth and roots	Teeth assessed (FDI notation or description); number of teeth or roots per group; roots per tooth measured	Free text; n
G. Imaging	Modality (CBCT, panoramic, periapical, cephalometric, micro-CT, digital model); device; kV, mA, exposure; voxel size; field of view; measurement software and version; superimposition or reference method	Free text; NR for each parameter not reported
H. Outcome definition	EARR metric (linear, volumetric, ratio, ordinal index); landmarks (e.g., incisal edge–apex, CEJ–apex, cusp tip–apex); unit (mm, mm^3, %, grade); definition of severe EARR (threshold or grading system); prevalence definition	Free text; NR
I. Examiners and reliability	Number of examiners; calibration; blinding to group and time point; reliability statistic (ICC, Dahlberg, kappa) with values and sample re-measured; interval between measurements	n; yes/no/NR; values
J. Analysis	Unit of analysis (patient, tooth, root, site); statistical tests; handling of clustering of teeth within patients (mixed model, GEE, averaging per patient, none); covariates or adjustment; sample-size calculation	Free text; none/NR
K. Results	Pre- and post-treatment values per group (mean ± SD or median [IQR]) per tooth type; change values; between-group difference with p-value or CI; prevalence and severity per group; declared primary EARR outcome (or, if none is declared, the principal EARR outcome of the abstract and results), with the direction of effect (e.g., CA < FA, no difference) and the number of significant between-group comparisons	Values as printed, with table/figure number; direction
L. Data-consistency checks (Section 2.6)	(a) Do reported change values equal post − pre values? (b) Do patient/tooth numbers agree across abstract, methods, tables and figures? (c) Do percentages match numerators and denominators? (d) Is there any statistical impossibility (CI > 100 %, p-values inconsistent with reported statistics)? (e) Overlap of methods text, acquisition settings or outcome values with another retrieved report or with a	Yes/No with numbers and locations; comparator RecID; decision: retain / exclude R16 / exclude R17 / record

Section	Items charted	Format / permitted values
	source cited by the report; (f) Declared shared cohort or trial registration shared with another report	
M. Reviewer notes	Any other observation relevant to the reliability of the EARR outcome (results only in figures, unclear units or denominators, discrepancies between sections)	Free text
