# Supplementary material for: External Apical Root Resorption with Clear Aligners Versus Fixed Appliances: A Scoping Review of Comparative Evidence, Treatment-Related Factors, Imaging Modalities and Consistency of Reported Data
Source: J Clin Med. 2026 Oct 22;15(21):8219. doi: 10.3390/jcm15218219 (PMC13764136; doi:10.3390/jcm15218219)
Supplement: Supplementary file 1 [file jcm-15-08219-s001.zip › Table_S9_Data-consistency_observations_R1.pdf]

Table S9. Observations on the consistency of reported data, recorded at full-text assessment, with the decision taken under the criteria of Section 2.6.

Sources: Full texts from PubMed Central and publisher sites and the PDFs held in the review's Zotero library. Checks: Internal numerical consistency (recalculation of differences from reported pre- and post-treatment values; consistency of sample sizes across text, tables and figures; percentages against denominators; dispersion relative to measurement error); overlap of methods text and outcome values with other retrieved studies and with their main cited sources; and shared cohorts stated in the reports. Values are quoted as printed in the articles. Reference numbers in square brackets refer to the manuscript reference list; reports not cited in the manuscript are identified by journal and DOI. All observations were made from the published reports; they describe the reported values only and imply no judgement about the quality of the reports or the conduct of the research.

(a) Reports excluded under the data-consistency criteria (criterion i, R16; criterion ii, R17), the report excluded as non-empirical (R14), and reports for which criterion (i) or (ii) was formally assessed and not met (retained)

Report	Decision	Evidence documented (with location)	Comparator publication
R1523 — Jyotirmay et al. 2021 [58]	Excluded, R16 (criterion i) and criterion ii	(i) CA-group values equal the FA-group values of Li et al. 2020 [12]: mean ARR 1.12 ± 1.34 mm; maxillary canine 1.53 ± 1.92 mm; maxillary lateral incisor 1.31 ± 1.33 mm; identical CBCT settings (10 × 10 cm FOV, 85 kV, 4 mA, 360°), same landmark, same criteria in paraphrase, same tests; Li et al. cited only in the Discussion; no shared cohort stated; different stated centre and device. (ii) Table 3: mandibular canine pre/post lengths identical in FA and CA (21.98 ± 1.71 → 20.96 ± 2.01) but different ARR (1.02 vs 1.21); FA total 21.63 → 20.42 = 1.21 mm vs reported 1.51 mm; CA mandibular central 18.26 → 17.61 = 0.65 vs reported 0.55; Table 4 counts 576 teeth per group vs 288 in Methods; 3/576 reported as 0.005%.	Li et al. 2020, Prog Orthod 21:1 (R0136)
R1630 — Gupta et al. 2023 [59]	Excluded, R16 (criterion i) and criterion ii	(i) Same sample-size basis as Almagrami et al. 2023 (1.53 ± 1.92 vs 0.14 ± 0.53 mm; G*Power 3.0.10; α 0.05; power 85%); same 5 inclusion and 8 exclusion criteria in the same order; identical CBCT parameters (120 kV, 5 mA, 230 × 170 mm; voxel "3 mm" vs 0.3 mm); same 3/6/9 mm levels, Sharpe grading, two-week blinded re-measurement; near-identical outcomes (inclination change −1.24 ± 8.11 / 4.63 ± 8.14 vs −1.23 ± 8.07 / 4.52 ± 8.03; OIIRR 0.63 / 0.32 vs 0.62 / 0.31 mm; prevalence 82 / 68% vs 82.5 / 68.5%); no shared cohort stated. (ii) CA root length 12.53 → 11.21 mm = 1.32 mm vs reported 0.32 mm; FA 11.83 → 11.25 = 0.58 vs 0.63; FA inclination 119.35 → 119.58 vs reported −1.24; identical palatal values in both groups; "p = 0.12" reported as significant.	Almagrami et al. 2023, Prog Orthod 24:6 (R0146)
R0499 — Alam et al. 2024 [57]	Excluded, R14 (non-empirical data)	Discussion states that the tabulated values are arbitrary, for illustrative purposes, and do not represent actual clinical data; Methods contain unfilled placeholders ("cite radiographic equipment and settings"); abstract results (0.2 / 0.1 / 0.05 mm) differ from full-text results (0.35 / 0.24 / 0.12 mm); Group C value after treatment lower than before yet reported as an increase.	—
R0258 — Su et al. 2026 [60]	Excluded, R17 (criterion ii); decision revised 26 September 2026 (Table S3, version 1.3)	Teeth per group stated as 224 (448 in total; abstract 488); Table 1 category counts sum to 342 (CA) and 320 (FA); Table 2 sums to 448 per group; "no resorption" percentages computed on 448, other categories on 224; CA upper-anterior moderate–severe stated as 30 teeth (17.14%) but Table 2 gives 12 + 12 = 24; 95% CI 92.65–107.35% for a proportion; re-measurement interval 2 days / 2 weeks / 20 days; reliability sample 5 vs 28 patients.	—
R0144 — Issrani et al. 2026 [61]	Excluded, R17 (criterion ii)	Signs of the model coefficients (β = −1.9; adjusted OR 0.62) are opposite to the direction of effect reported in the text.	—
R0307 — Ay Ünüvar et al. 2025 [62]	Excluded, R17 (criterion ii)	Per-tooth medians reported for an ordinal 0–3 score (e.g., 0.05; 0.15 [0.00–0.25], Table 2) are not possible values of that score; totals 0.40 vs 1.00 reported without a test; announced within-group comparisons not reported.	—
R0202 — Zhou et al. 2025 (BMC Oral Health; 10.1186/s12903-025-05939-x)	Excluded, R17 (criterion ii)	Self-controlled design (period without vs with micro-osteoperforation in the same patients), CBCT. Reported "mean resorption" 2.13 vs 1.96 mm, whereas canine root length changes from 13.60 to 13.26 mm (Δ 0.34 mm); resorbed volumes of about 50 mm³ in three months; SD of 0.04–0.09 mm for root lengths.	—
R0291 — Cheng et al. 2026 [53]	Retained (Question 1); criterion (ii) assessed, not met; decision revised 27 September 2026 (Table S3, version 1.4)	The between-group result for the primary comparison is stated in Results 4.1 and Figure 4 (length change: no significant difference among aligners, fixed appliances and fixed appliances with miniscrews; central-incisor volume change greater with fixed appliances and miniscrews, p < 0.0001); values are given in figures only. Recorded discrepancies: the Abstract reports no significant between-group differences and a greater volume change of central than lateral incisors, whereas Section 4.2 reports no significant central–lateral difference; volume-change categories sum to 91%.	—

Report	Decision	Evidence documented (with location)	Comparator publication
R0493 — Pham et al. 2025 (Open Dent J; 10.2174/0118742106363883250203060336)	Retained (Question 2); criterion (ii) assessed, not met; decision revised 27 September 2026 (Table S3, version 1.4)	Primary outcome identifiable in Table 1: root-length change −0.46 ± 0.33 mm (i-PRF) vs −0.80 ± 0.54 mm (control), p = 0.004. Recorded discrepancies: the results call the difference significant, whereas the abstract and conclusion call it non-significant; sex reported as 9 M + 22 F and 9 M + 21 F. Same institution (Can Tho) and sample size as R0473 (Le et al. 2024, retained); different intervention and different outcome values.	R0473
R0577 — Abd ElMotaleb et al. 2024 (Sci Rep; 10.1038/s41598-024-58268-4)	Retained (Question 2); criterion (ii) assessed, not met; decision revised 27 September 2026 (Table S3, version 1.4)	Root resorption (a secondary outcome) measured on CBCT and compared between groups, with values per group in the results table. Recorded discrepancies: CBCT analysis in 18 patients (“36 CBCT images … 18 pre-retraction and 18 post-retraction”) of 32 randomised, without a reported reason and with the abstract stating that no patients dropped out; per-group split of the 18 not reported; a Kruskal–Wallis test named for two groups; a date given in place of a registration number.	—
R0808 — Prasad et al. 2025 (J Pharm Bioallied Sci; 10.4103/jpbs.jpbs_607_25)	Excluded, R17 (criterion ii)	Extraction vs non-extraction treatment in 130 patients with root-filled teeth (zirconia crowns) and contralateral vital teeth; CBCT. Methods define both scans as taken “at the beginning” and “at the commencement” of treatment, so the time point of the values reported as post-treatment cannot be established; Table 2 (extraction group, vital teeth): 107.80 − 104.33 = 3.47 vs reported difference −3.55; the same p-values (0.975; 0.136) are reported for the extraction–non-extraction comparison of two different outcomes (linear ERR, Table 1; root area, Table 2); root area labelled “mm” in Table 2 and mm² in the methods; significance threshold stated as p ≤ 0.005; numbers of teeth per group and unit of analysis not reported.	—
R1159 — Seker et al. 2020 (Bezmialem Science; 10.14235/bas.galenos.2019.3658)	Excluded, R17 (criterion ii)	Crown Herbst vs Twin Block on panoramic radiographs. Table 5 (Twin Block, T2) repeats values of Table 4 (Herbst, T1): length of tooth 36 (261.6 ± 60.2) and areas of 36/35/34/33/25; area of 26 at T2 = 14422.8 ± 21492.8; units not stated (probably pixels).	—
R1297 — Mahmoud et al. 2025 (J Orthod Sci; 10.4103/jos.jos_114_25)	Retained (Question 2); criterion (ii) assessed, not met; decision revised 27 September 2026 (Table S3, version 1.4)	Primary outcome (linear and volumetric root resorption per group) identifiable in Tables 3 and 4 (e.g., overall root-length change −0.97 ± 0.02, −0.93 ± 0.05 and −0.81 ± 0.03 mm). Recorded discrepancies: standard deviations of 0.02–0.07 mm for changes measured on CBCT with a voxel size of 0.2 mm in groups of seven patients; one positive value (0.98) among negative changes in Table 3; volumes labelled “mm”; a 1:1 allocation ratio stated for three groups; SPSS version 18 (abstract) and 23 (methods).	—
R1559 — Kaur et al. 2020 (J Dent Spec; 10.18231/j.jds.2020.002)	Retained (Question 2); criterion (ii) assessed, not met; decision revised 27 September 2026 (Table S3, version 1.4)	Primary outcome (mean root resorption score per group) identifiable in Table 2 (e.g., upper anterior 0.40 ± 0.19 non-extraction vs 0.44 ± 0.21 extraction). Recorded discrepancies: Mann–Whitney Z = −6.09 and r = 0.802 incompatible with these values; ICC with 79 degrees of freedom for 20 patients; received 15 October 2020, accepted 11 February 2020; non-extraction treatment duration printed as “14.4 years”.	—
R1656 — Alshayea et al. 2020 (J Res Med Dent Sci 8(5):101–109; no DOI)	Retained (Question 2); criterion (ii) assessed, not met; record identified at the repeated deduplication (Table S3, Part C)	(ii) Table 1: mean EARR values of about 2 mm per tooth type on panoramic radiographs (e.g. upper central incisor 2.02 ± 1.25 mm; upper lateral incisor 1.85 ± 0.96 mm) are not compatible with the printed grade distributions (55.6% and 48.1% of teeth in grade 0; grade 3 defined as > 2 mm); the "Total" row reports grade percentages on a denominator other than the 540 teeth stated; Table 3: identical SDs (1.78 mm) for the extraction and non-extraction groups (72 and 48 teeth). The primary outcome (EARR in mm by treatment factor, Tables 2, 3 and 5) can be identified (rule b).	—
R0701 — Tahir et al. 2025 (Br Dent J; 10.1038/s41415-025-8887-9)	Retained (Question 2); criterion (ii) assessed, not met; report obtained and assessed at full text during the revision (October 2026; Table S5)	Root resorption is a secondary outcome. The EARR outcome is identifiable in Table 3 (root length per group before and after treatment; e.g., mandibular central incisors, medians 15.2 → 14.8 mm with interproximal reduction and 14.4 → 12.8 mm with mandibular incisor extraction; post-treatment p = 0.042). Abstract: “MIE resulted in greater root resorption (median difference: 2.0 mm; p = 0.042)”; 2.0 mm equals the difference between the post-treatment lengths (14.8 vs 12.8 mm, Table 3), whereas the pre- to post-treatment changes calculated from the printed medians are 0.4 and 1.6 mm. Abstract: ABO-OGS score “higher in the IPR group (38.20 …) than the MIE group (43.50 …)”.	—
R1624 — Kurian et al. 2025 (Bioinformation; 10.6026/973206300210495)	Excluded, R17 (criterion ii)	Retrospective series of 100 “cases” (46 endodontically treated and 54 vital teeth) under orthodontic forces; CBCT; appliance not reported. Percentages reported with two decimals are incompatible with the stated denominators: endodontically treated 46.21% and vital 53.79% vs 46/100 and 54/100; age groups 54.12 / 27.34 / 18.54%, sex 57.26 / 42.74%	—

Report	Decision	Evidence documented (with location)	Comparator publication
		and tooth types 38.21 / 32.44 / 29.35% are not proportions of 100 cases; complication rates (7.8 / 5.3, 13.6 / 11.2, 3.2 / 2.1%) do not correspond to integer counts of 54 or 46 teeth; resorption reported only for incisors and molars although premolars formed the largest group (38.21%), without subgroup sizes; unit of analysis (case vs tooth) undefined.	
R0501 — Al-Rokhami et al. 2025 [64] and R0643 — Al-Rokhami et al. 2026 [65]	Excluded, R16 (criterion i)	Same first author and two examiners (R.K.A., K.A.S.), identical training/reliability description (8-h training, 20 practice scans, 15% re-measured after 2 weeks), same protocol (H1–H3 at 2/4/6 mm, Invivo 6), overlapping recruitment (R0259: 2015–2024; R0501, R0643: 2015–2022); no shared patients stated. FA extraction group of R0259 vs extraction group of R0501: CBW-mid H1 T1 0.96 ± 0.17 in both; CBW-mid H2 0.84 ± 0.13 → 0.85 ± 0.14 vs 0.85 ± 0.14 → 0.88 ± 0.14; IC width H1 T1 3.73 ± 0.87 vs 3.72 ± 0.89; IC width H3 3.03 ± 0.81 → 3.23 ± 0.96 vs 3.01 ± 0.78 → 3.31 ± 0.96. CA group of R0259 vs CA extraction group of R0643: CBW H1 T2 0.58 ± 0.40 vs 0.59 ± 0.42; CBW-mid H2 T1 0.98 ± 0.21 in both; apical resorption 0.87 ± 0.77 vs 0.91 ± 0.80 mm; root length 21.29 ± 2.04 → 20.42 ± 2.20 vs 21.51 ± 2.06 → 20.60 ± 2.32. CA non-extraction group of R0643 (n = 60) vs CA group of R0293 (n = 60): identical values in both abstracts (apical resorption 0.32 ± 0.56 mm; decrease in root–canal distance 0.40 ± 0.58, 0.47 ± 0.78 and 0.30 ± 0.98 mm at H1–H3). Discordant details: CBCT KaVo (R0259, R0501) vs i-CAT (R0643); IRB 2022065 vs LZUKQ-2020-20 vs not stated. (ii) R0501 Table 2: reported mean changes do not equal T1–T2 differences (U1 volume extraction 476.07 → 451.62 = 24.45 vs reported 28.78; IC volume extraction 116.31 → 108.73 = 7.58 vs 40.15). Earliest report of the cohort: R0268 (see below). Related reports: R0321 (excluded, R05), R0293 (excluded, R16), R0497 (excluded, R05).	R0268; R0259 [63]; R0293 [66]
R0293 — Al-Rokhami et al. 2026 (Odontology) [66]	Excluded, R16 (criterion i)	Direct CA–FA comparison in non-extraction incisor retraction (CA n = 60, FA n = 44; 208 central incisors; Zhenjiang; 2015–2023). The FA arm reports values identical to those of the entire sample of R0321 (Al-Rokhami et al. 2025, Int Orthod [67]; 44 patients, 88 central incisors, 18 M / 26 F, 2015–2023, same centre, same examiners' description): sex-weighted U1 length from R0321 Table II, T1 (18 × 22.04 + 26 × 21.12)/44 = 21.50 mm and T2 (18 × 20.59 + 26 × 20.42)/44 = 20.49 mm, equal to the FA values of R0293 Table 2 (21.50 ± 1.35 → 20.49 ± 1.41 mm); IC width H1 (18 × 4.29 + 26 × 3.86)/44 = 4.04 → (18 × 4.04 + 26 × 3.56)/44 = 3.76 mm, equal to R0293 (4.04 ± 0.68 → 3.76 ± 0.63); mean root resorption 1.01 ± 0.68 (R0321) vs 1.01 ± 0.74 mm (R0293). No shared cohort stated; R0321 not cited in R0293. R0321 was published first (online 18 March 2025 vs 29 May 2025) and was retained for assessment (excluded on concept grounds, R05). Additional observations: R0293 Table 1 gives the CA treatment duration as 2.02 ± 7.90 years; R0321 Tables VI and VII are headed "n = 120 centrals" for a sample of 88.	R0321 — Al-Rokhami et al. 2025 (Int Orthod) [67]; R0643 [65]
R0268 — Al-Rokhami et al. 2025 (J World Fed Orthod; 10.1016/j.ejwf.2025.05.005)	Excluded, R05 (patient-level factors only); earliest report of the Zhenjiang extraction cohort, retained for assessment under the rule of Section 2.6	Extraction FA cohort (42 patients, 84 central incisors, Zhenjiang, 2015–2022) analysed by sex, age and skeletal classification; available online 3 July 2025. Sample size, period and centre match the extraction group of R0501 (Front Physiol 2025 [64]; extraction n = 42, 2015–2022), whose values are near-identical to those of the FA extraction group of R0259 [63] (Sci Rep, 10 July 2025); no shared cohort stated in any of them. Internally consistent (subgroup counts sum to 42; abstract and Table 5 values agree once order and sign are reconciled).	R0501 [64]; R0259 [63]
R0259 — Al-Rokhami et al. 2025 [63]	Excluded, R16 (criterion i); decision revised 27 September 2026 (Table S3, version 1.4)	Direct CA–FA comparison in first-premolar extraction cases (33 CA / 33 FA; Zhenjiang; 2015–2024). Its FA extraction group reports values near-identical to those of the extraction group of R0501 [64], and its CA group values near-identical to those of the CA extraction group of R0643 [65] (see above); the cohort was first reported in R0268 (available online 3 July 2025), a week before R0259 (10 July 2025). No shared cohort stated. Additional observation: the fixed appliance is named as Victory Series (3M Unitek) and, in the following paragraph, as 0.022 × 0.028-in Damon passive self-ligating brackets; proximity percentages labelled per 132 incisors are computed on 396 sites.	R0268; R0501 [64]; R0643 [65]
R0529 — He et al. 2025 (AJODO) [54]	Excluded, R16 (criterion i); decision revised 27 September 2026 (Table S3, version 1.4); earlier report R0862 [47] retained	Same institution (Shanghai Ninth People's Hospital), overlapping author team, identical ethics approval number (SH9H-2024-T42-1) and group sizes (30 CA / 30 FA), both in orthodontic–orthognathic treatment of skeletal Class III, with mutually exclusive inclusion criteria (R0529: bilateral maxillary first-premolar extraction; R0862: no extraction except third molars). The cephalometric tables of the two reports (R0529 Table II; R0862 cephalometric table) give identical values for three of twelve variables in both groups at T0, T1 and T2: interincisal angle FA 128.42 ± 8.8, 130.55 ± 9.29, 129.5 ± 9.7° and CA 129.63 ± 9.04, 135.49 ± 8.83, 131.98 ± 9.62° (p = 0.647 and 0.055 in both); overjet FA 2.24 ± 1.97, 7.33 ± 3.67, 2.54 ± 0.71 mm and CA 0.97 ± 2.36, 5.60 ± 2.94, 2.50 ± 0.64 mm (identical in absolute value only: printed with negative signs at T0 and T1 in R0862); overbite FA 0.07 ± 2.28, 1.18 ± 2.60, 1.59 ± 1.03 mm and CA 0.19 ± 1.69, 0.89 ± 1.55, 2.09 ± 1.08 mm	R0862 [47]

Report	Decision	Evidence documented (with location)	Comparator publication
		(identical in absolute value only: printed with negative signs at T1 in the FA group and at T0 in the CA group in R0862). No shared cohort stated. R0862 was available online on 22 October 2024; R0529 was accepted in December 2024. Additional observations for R0529: Abstract root-length reduction 1.09 ± 0.70 (CA) and 1.29 ± 0.73 mm (FA) vs 1.15 ± 1.10 and 1.46 ± 1.12 mm in Table V and the Discussion; FOV printed as "16 × 13 mm".	
R0863 — Almagrami et al. 2025 (AJODO) [48]	Retained; criterion (i) checked, not met	Same first author as R0146 (Almagrami et al. 2023 [40]) and the same institution (First Affiliated Hospital of Zhengzhou University) and ethics approval number (2022-KY-0800-001); mutually exclusive samples (80 patients, Class II Division 1, four-premolar extraction, treatment period NR vs 40 patients, non-extraction); one identical pair of baseline values, in different groups (1.99 ± 1.68 mm, total central-incisor bone thickness: CA in R0146, FA in R0863); no demographic value in common; R0146 and R0809 cited as comparators only. One reconcilable discrepancy (Table S9(b)).	R0146 [40]
R0053 — Cha et al. 2021 (AJODO; 10.1016/j.ajodo.2020.06.043) and R0062 — Li et al. 2025 (Clin Oral Investig) [55]	Both retained; criterion (i) checked, not met	Same institution (China Medical University, Shenyang) and corresponding author; recruitment periods overlap (2015–2019 vs January 2017–December 2023); different teeth (maxillary incisors vs paired maxillary molar palatal roots), factors (dehiscence/fenestration, extraction and duration vs sinus–root relationship, extraction site and appliance) and outcome values. No shared cohort stated; R0053 not cited in R0062.	Each other
R0473 — Le et al. 2024 (J Int Soc Prev Community Dent; 10.4103/jispcd.jispcd_5_24)	Retained; criterion (i) checked, not met; criterion (ii) assessed, not met	Same institution (Can Tho University of Medicine and Pharmacy) and sample size (31 patients undergoing maxillary canine retraction) as R0493 (Pham et al. 2025, retained; criterion (ii) assessed, not met); different interventions (NiTi closed-coil spring vs elastomeric chain; injectable PRF vs control) and different outcome values; no shared cohort stated. (ii) Table 3: the SD column of the between-side differences (0.878, 0.917, 0.72, 0.514) repeats the p-value column (0.878, 0.917, 0.723, 0.514); Table 6 prints identical lingual values for both sides (−0.97 ± 0.21, −0.39 ± 0.98, −0.79 ± 1.09) where Tables 4 and 5 give −1.03, −1.37 and −4.07 for the coil-spring side; the U3–PP angle change is given in mm. Primary outcome (root length 15.44 → 14.52 vs 15.35 → 14.45 mm; p = 0.878) identifiable.	R0493
R1463 — Gupta et al. 2023 (Dental Press J Orthod; 10.1590/2177-6709.28.5.e232388.oar)	Excluded, R16 (criterion i); earliest report R0624 retained	Same cohort as R0624 (16 subjects, 9 M / 7 F, 17–25 years, 21.85 ± 2.45, NiTi 150 g, 5 months, same authors) without a shared cohort stated; R0624 was available first (online 13 June 2023).	R0624 — Gupta et al. (APOS Trends Orthod 2024;14:156–163)
R0683 — Al-Somairi et al. 2025 [41]	Retained; criterion (i) checked, not met	Different institution (China Medical University, Shenyang; treatment 2017–2024, vs First Affiliated Hospital of Zhengzhou University) but the same CBCT settings (120 kV, 23 × 17 cm, 0.3 mm) and same sample-size basis (0.14 ± 0.53 vs 1.53 ± 1.92 mm) as Almagrami et al. 2023 [40]; no outcome values in common; no shared cohort stated. Minor: sex row "12 (40.0) / 18 (30)"; narrative direction of two extraction-group bone changes opposite to printed values; "(> 0.05)" in Results vs "< 0.05" in Abstract for canine/premolar findings.	Almagrami et al. 2023 [40]
R0146 — Almagrami et al. 2023 [40]	Retained; criterion (ii) assessed, not met (reconcilable)	FA apical resorption 0.68 ± 0.97 mm (abstract; equals the FA central-incisor value in the results), 0.62 ± 0.78 mm (results, between-group), 0.62 ± 0.54 mm (discussion); prevalence 83/69% (results) vs 82.5/68.5% (discussion); "p < 0.000"; ICCs 0.97 and 0.92 described as "non-significant". Primary value identifiable in the results.	—

(b) Discrepancies recorded in retained reports (Questions 1 and 3) that did not meet the exclusion criteria

Report	Criterion	Observation (with location)	Handling
R1602 Pansotra 2026 [38]	observation	With the printed means, SDs and n = 20 per group, the stated independent t-tests give p ≈ 0.00006 (maxillary) and p ≈ 0.0002 (mandibular), not the reported p = 0.003 and p = 0.01; direction and significance unchanged; examiners, reliability, device and landmarks not reported; ethics committee not named	Record in Table S9; retention consistent with criterion (ii)
R0489 Mertoglu 2025 [36]	observation	Tooth 32 CA change +0.11 ± 3.83 mm (SD larger than for the other teeth, 0.45–0.97) with p = 0.04, whereas a t-test on the printed values gives p ≈ 0.24; tooth 12 reported p < 0.001 vs ≈ 0.004 recomputed; test per tooth not specified (t or Mann–Whitney), so not conclusive	Record in Table S9
R0169 Toyokawa-Sperandio 2021 [34]	observation	"A single examiner performed all measurements" yet inter-examiner ICCs (0.82–0.94) are reported; between-group difference for tooth 21 stated as 0.35 mm, printed means give 0.34 mm	Record

Report	Criterion	Observation (with location)	Handling
R0136 Li 2020 [12]	observation	Abstract: 373 roots from 70 patients, whereas Table 5 gives 373 CA and 369 FA teeth (742; 10.6 per patient); FA prevalence 82.11% vs 81.84% implied by the Sharpe grade distribution	Record
R0361 Alassiry 2022 [13]	observation	Per-tooth grade counts in Table 1 sum to OPG 54/50/12/4/0 and CBCT 29/67/17/7/0, whereas Table 2 marginals (and text) give 47/60/12/1/0 and 20/80/18/2/0; tooth 22 labelled "right" lateral incisor	Record; Q3 study
R0087 Bespalez-Neto 2026 [35]	observation	Compliance categories sum to 95%; metric described as volumetric in Introduction but is a linear surface distance	Record
R0596 Withayanukonkij 2023 [16]	observation	Standard deviations of the change values (0.02–0.06 mm for CA, 0.06–0.16 mm for FA) are of the same order as the Dahlberg error (0.02–0.03 mm); T0/T1 differences match reported changes within rounding	Record; retained
R0636 Darvizeh 2026 [44]	observation	CA–FA comparison rests on the 11-patient-per-group test set of an AI-validation study; authors declare no overlap with their meta-analysis dataset	Record; retained
R0296 Husain 2024 [43]	observation (reconcilable)	Table 3: for tooth UL3 the printed T1 value is lower than T2 in all three groups (e.g. FA 18.73 → 23.22 mm), opposite to UR3 (23.19 → 18.75) — apparently transposed columns; baseline root volumes of IBR lateral incisors (37.2 mm³) are far below those of the other groups (≈ 60 mm³); narrative IBR-vs-INBR volume p < 0.001 whereas Table 4 gives 0.002–0.041; FA mean root-length reductions of 1.8–4.4 mm are far larger than in any other comparison while volumetric loss equals that of aligners; the text reports no volumetric difference between the fixed-appliance and aligner groups, whereas Table 4 gives significant differences between fixed appliances and aligners with bite ramps for root volume (4 of 6 teeth) and percentage volume loss (3 of 6).	Record; retained — primary outcome identifiable from Table 3; column transposition reconcilable
R1638 Mai 2021 [37]	observation	No resorption values printed in text or tables (Figures 2–7 only); captions of Figures 5 and 7 state "P > 0.05" and mark "* P < 0.05" for the same comparison, differing from the results text; “randomly divided” stated in the abstract only, the methods describing a recruited cohort; single examiner without reliability statistic; the 0.3 mm error is cited from the literature, not measured; CBCT parameters not reported	Record; retained — overall result (no difference) identifiable
R0862 He et al. 2025 (JWFO) [47]	observation	Table 5: phase changes T0–T1 (FA 0.39 ± 0.88; CA 0.28 ± 0.83) and T1–T2 (0.37 ± 0.62; 0.24 ± 0.75) do not sum to the printed T0–T2 changes (0.64 ± 0.8; 0.4 ± 0.79); recalculation from the group means of Tables 3 and 4 (FA 11.33 → 10.95 → 10.58) gives 0.75 mm for T0–T2; between-group test described as t / Mann–Whitney in the methods and one-way ANOVA in the Table 5 footnote. Three cephalometric variables identical to those of R0529 (Table S9(a)).	Record; retained as the earlier of the two reports; direction identifiable
R0057 Al-Gumaei et al. 2025 [46]	observation	Voxel size stated as 0.25 mm and, in the next sentence, 0.3 mm; the reliability check is described as intra-rater (methods) and inter-rater (statistical analysis); baseline comparability stated as “no significant differences (P < 0.05)”. Group sizes and Table 3 subgroup counts (13 + 17 + 30; 20 + 24 + 16) reconcile.	Record; retained
R0863 Almagrami et al. 2025 [48]	observation	FA central-incisor root length 22.97 → 21.03 mm (Table III) = 1.94 mm vs 1.86 ± 1.50 mm in Table IV; other Table III/IV pairs reconcile (e.g., FA central-incisor volume 260.31 − 233.26 = 27.05 vs 27.06). Same first author as R0146 (Almagrami et al. 2023 [40]) and the same institution and ethics approval number; mutually exclusive samples; one identical pair of baseline values, in different groups (Table S9(a)); R0146 cited as comparator only.	Record; retained — criterion (i) checked against R0146, not met
R0040 Zhang et al. 2026 [50]	observation	Supplementary Table IV: horizontal displacement of the incisal edge printed as −5.91 ± 2.68 (FA) and 6.42 ± 2.25 (CA) with p = 0.381 (sign of one value); the GEE mean difference in rRCR is printed as −4.27% with FA as reference, whereas the printed marginal means give CA − FA = +4.27%. Grade distributions and group totals (240 / 570 / 270 teeth) reconcile.	Record; retained
R0809 Chen et al. 2023 [45]	observation	Table III pre/post values reproduce the Table IV changes (e.g., CA volume 283.33 − 259.65 = 23.68); group sizes consistent. Linear tooth-length change NS (p = 0.976) while volumetric loss differs (p = 0.030): the direction of the comparison depends on the metric.	Record; retained
R0379 Bahaj et al. 2026 [49]	observation	Recalculated T1–T2 differences reproduce the printed changes to within 0.01 mm (e.g., TT type I second premolar 16.24 − 15.64 = 0.60 vs −0.60 ± 0.51); sample sizes consistent (4 × 20). Treatment duration differs between appliances by 9–15 months and is not adjusted for.	Record; retained
R0865 Sabban et al. 2026 [51]	observation	Presence of resorption printed as 49 (84.5%) of 57 fixed-appliance patients (49/57 = 86.0%), whereas the severity and laterality rows imply 49 and 48 patients with resorption and, for aligners, 23 and 25 against 24 in the presence row; the ≥ 3 mm category is called “severe” in the methods and “moderate-to-severe” in the results, and the discussion gives 22.8% and 5.2% of resorption cases; aligner-group age 26.80 ± 9.21 years in the table and 26.8 ± 19.5 in the abstract; treatment-duration difference reported with p = 0.005 (table) and p = 0.026 (discussion); no reliability statistic.	Record; retained — primary outcome (patient-level prevalence) identifiable

Report	Criterion	Observation (with location)	Handling
R0905 Liu et al. 2025 [52]	observation	Single clinician; no reliability statistic; appliance groups of 56 FA and 13 CA patients without baseline characteristics by appliance; correlation between age and the root-filled–vital difference reported as r = 0.8362 with p = 0.0254 for n = 69 (not reproducible); sample size supported by a post hoc power calculation.	Record; retained
R0291 Cheng et al. 2026 [53]	observation	See Table S9(a): between-group values in figures only; abstract states no significant between-group differences whereas results section 4.1 reports greater central-incisor volume loss with fixed appliances and miniscrews (p < 0.0001); volume-change categories sum to 91%; examiners and reliability not reported.	Record; retained — direction identifiable from Results and Figure 4

(c) Location of the two reports with identical values (Li et al. 2020 [12]; Jyotirmay et al. 2021 [58]) in the previous syntheses that pooled both (manuscript Section 4.3)

Synthesis	Reference numbers in the synthesis (Li; Jyotirmay)	Tables (row labels as printed)	Pooled analyses (figures and tables; row labels as printed)	Identical values printed in the synthesis
Singh et al. 2024 [5]	17; 16	Table 2: "None et al, 2021" and "Yi et al, 2018". Table 3: "None J et al, 2021" (CA 1.12 ± 1.34 mm) and "Yi et al, 2020" (FA 1.12 ± 1.34 mm). In both tables, the data of Li et al. 2020 appear under the label "Yi et al." and are linked to reference 14 of the synthesis (Yi et al. 2018)	Figure 4 (maxillary anterior teeth) and Figure 5 (mandibular anterior teeth): "Jyotirmay et al,2021" and "Yuan Li et al,2020"	Table 3: 1.12 ± 1.34 mm. Figure 4: 1.23 ± 1.31, 1.31 ± 1.33 and 1.53 ± 1.92 mm (Jyotirmay CA; Li FA)
Darvizeh et al. 2025 [6]	14; 24	Table S4 (Supplementary Materials): "Characteristics of included studies in this systematic review and meta-analysis"	Figure 4 (top panel, mm; bottom panel, %), Figures 5 and 6 (incisor subgroups), Figure 9, Figure 10 (12–24 months subgroup) and Figure 11 (CBCT subgroup): "Jyotirmay et al 2021" and "Li et al 2020"	Figures 5 and 6, subgroup "MxL": 1.31 ± 1.33 (Jyotirmay CA; Li FA)
Yıldırım and Topbas 2026 [7]	16; 18	Table 1: "Li et al., 2020" and "Jyotirmay et al., 2021". Table 3: "Li 2020" (FA 1.12 ± 1.34 mm) and "Jyotirmay 2021" (CA 1.12 ± 1.34 mm)	Figure 2 (overall) and Figure 3 (non-extraction/mixed subgroup)	Table 3: 1.12 ± 1.34 mm (Li FA; Jyotirmay CA)
Samulak et al. 2026 [9]	10; 29	Table 2: "Li et al. 2020" (FAT 1.12 ± 1.34) and "Jyotirmay et al. 2021" (CAT 1.12 ± 1.34)	Figure 2; Table 5, subgroup "Extraction cases included" (these two reports only)	Table 2: 1.12 ± 1.34 mm (Li FA; Jyotirmay CA)
Selvaraj et al. 2025 [10]	11; 31	Supplementary Table 3 (descriptive data of the quantitative analysis)	Section 4.2 and Figure 4 (a)–(f): "Li Y 2020" and "Jyotirmay 2021"	Figure 4 (a), (b) and (e): 1.23 ± 1.31, 1.31 ± 1.33 and 1.53 ± 1.92 mm (Li FA; Jyotirmay CA)
Butsabul et al. 2024 [68]	19; 18	"Table – Characteristics of the included studies" (Table 1 in the text): "Jyotirmay et al." and "Li et al."	Figure 2 and Figure 3 (A–G), aligner arms only: "Jyotirmay 2021" and "Li 2020"	Not applicable: only aligner arms were pooled, so the shared values enter once, as the aligner data of Jyotirmay et al.

Part (c): Row labels and values are reproduced as printed in each synthesis, without the reference numbers attached to the labels (these are given in the second column). CAT and FAT, clear aligner and fixed appliance treatment (labels in [9]); MxL, maxillary lateral incisor (label in [6]). Sources: Full texts of the syntheses.

ARR, apical root resorption; CA, clear aligners; CBW, cortical bone width; CI, confidence interval; FA, fixed appliances; IC, incisive canal; ICC, intraclass correlation coefficient; i-PRF, injectable platelet-rich fibrin; L-PRF, leukocyte- and platelet-rich fibrin; OIIRR, orthodontically induced inflammatory root resorption; OR, odds ratio; SD, standard deviation.
